# Spatial richness of neural magnetic fields

**DOI:** 10.1371/journal.pcbi.1014283

**Published:** 2026-05-22

**Authors:** Ziad Ali, Ada S. Y. Poon

**Affiliations:** Electrical Engineering Department, Stanford University, Stanford, California, United States of America; The Open University of Israel, ISRAEL

## Abstract

Brain implants that measure neural magnetic fields, rather than electrical potentials, are expected to confer significant clinical advantages related to implant longevity and signal fidelity due to the elimination of the electrode-tissue interface. However, the informational differences between neural electrical potentials and magnetic fields remain poorly understood. Using a mathematical formalism based on neuronal current sources, we directly establish the complementary informational content of extracellular magnetic fields and electrical potentials. This formalism also reveals that extracellular magnetic fields generated by spiking neurons inherently exhibit one order lower spatial polarity than electric fields, resulting in more favorable distance-scaling characteristics. We then use computational modeling to illustrate how dense networks of neurons are easier to distinguish and spike sort on the basis of their magnetic, rather than electrical, spike templates. Lastly, we show how the solenoidal nature of neural magnetic fields facilitates approximate morphological reconstruction, even with sparse sensor arrays. Our findings highlight the unique experimental advantages of neural magnetic field sensing, motivating the development of compact, low-noise devices capable of meeting the stringent sensitivity requirements for cortical recordings.

## Author summary

Microelectrode arrays are the gold standard for measuring single-cell neural activity, but they face key challenges that limit their clinical and informational utility. Magnetic sensing is expected to alleviate many of these clinical challenges by eliminating the sensor-tissue interface, but the informational content of neural magnetic fields is not well understood. We demonstrate how the origin of neural magnetic fields in cellular longitudinal currents results in the formation of spatially rich extracellular spike templates that are more reflective of neuronal morphology than extracellular electrical potentials. This spatial richness enables dense and highly-populated networks of cells to be more easily distinguished on the basis of their magnetic field measurements, using techniques such as spike sorting, which can enhance the accuracy of neural decoding. The unique solenoidal and morphological properties of neural magnetic fields also facilitate new techniques, such as morphological reconstruction. Our results demonstrate the untapped informational potential of cellular-scale neural magnetic fields, and should motivate the development of micro-scale magnetic sensors capable of measuring them.

## 1 Introduction

Over the past two decades, researchers have achieved significant breakthroughs in decoding neural representations of cognitive intentions, such as limb movements [1] and speech [2], as well as external stimuli, including visual [3] and auditory [4] signals. These advances, which are foundational to the development of next-generation neuroprosthetics, critically depend on specialized hardware capable of precisely recording the activity of hundreds to thousands of densely packed neurons simultaneously. Implantable microelectrode arrays (MEAs) remain virtually the only clinically deployed technology capable of enabling such recordings, with devices such as the Utah array [5] and Neuropixels probe [6] widely adopted for single-cell resolution recordings in human and primate cortices.

Nonetheless, implantable MEAs face several significant challenges and limitations. One critical issue is the requirement for direct tissue contact, essential for providing conductive paths for current flow between the electrodes and target neurons. This requirement reduces the long-term efficacy of chronically implanted arrays because of gliosis, an immune response resulting in scar tissue formation around foreign bodies. Such scar tissue encapsulates the implant, impeding current flow [7]. Consequently, implants often fail in less than a few years [1,8,9]. Moreover, the metal-solution interface of microelectrodes forms a capacitive double-layer effect, leading to charge screening that negatively affects signal quality [10]. From a device perspective, another major limitation of electrodes is the tradeoff between sensitivity and selectivity. Smaller electrodes improve spatial resolution, but their heightened electrical impedance results in worse thermal noise and signal loss [11]. In addition, since microelectrodes measure electrical potential differences, all arrays must include a reference electrode. Optimizing the size and placement of this electrode is a non-trivial problem and can degrade signal-to-noise ratio (SNR) [12–14].

Magnetic-field sensing, unlike electrical-potential sensing, does not depend on a conductive path between sensor and tissue and is reference-less, making it a promising approach to overcome the aforementioned limitations. When neurons spike, they generate small magnetic fields in much the same way that current-carrying wires generate rotating magnetic fields according to Maxwell’s equations [15]. Since the magnetic permeability of tissue is essentially the same as air, these fields propagate undistorted throughout the brain and even outside the body, unaffected by the dielectric properties of tissue which filter electric currents [16]. These clinical advantages have been partially realized at the non-invasive level in magnetoencephalography (MEG), a contact-less technique that eliminates the sensor-skin impedance problems common in electroencephalography (EEG) [17,18]. For some analyses, MEG and EEG are known to capture complementary information, such as in interictal spike localization for temporal lobe epilepsy, in which MEG’s greater specificity is offset by EEG’s wider sensitivity [19–21]. However, MEG is increasingly demonstrating informational *advantages* over EEG, such as in [22], in which MEG facilitated brain-to-text decoding that was intractable with EEG. It is generally believed that these advantages stem from enhanced spatial resolution due to a lack of dielectric smearing; however, the *intrinsic informational quality* of the magnetic fields relative to the electrical potentials at the cellular level has been underexplored.

We believe that, irrespective of tissue dielectrics, multi-magnetometer arrays (MMAs) that measure activity at the *single-cell level* are likely to exhibit informational benefits over MEAs, in addition to their known clinical advantages, due to the unique, fundamental properties of neural magnetic fields. However, the deployment of magnetic sensing at the cellular level in neuroscience remains constrained by the extremely weak magnitude of neural magnetic fields—typically in the low picotesla to femtotesla range—relative to the noise floor of current microscale sensor technologies [23]. Existing demonstrations have primarily focused on obtaining low-SNR signals from large neurons or aggregated cellular activity, requiring extensive averaging to extract meaningful data [24–27]. Single-shot magnetic recording at the level of individual cortical neurons has yet to be achieved.

To validate our hypothesis that MMAs exhibit *informational* advantages over MEAs, we rely on theoretical and computational frameworks to evaluate the informational content of neural magnetic fields at the cellular scale. First, we present a rigorous mathematical formulation describing how spiking neurons generate extracellular electrical potentials and magnetic fields, showing that these signals arise from fundamentally distinct current sources: transmembrane currents for electric fields and longitudinal currents for magnetic fields. From this distinction, three key insights follow: for morphologically realistic cells, electrical potentials and magnetic fields convey complementary information about the spiking neuron and its activity; they scale differently with distance from the source; and they exhibit distinct vector field properties—curl-free for electric fields versus divergence-free for magnetic fields. Building on these insights, we investigate, at both the single-cell and network scale, how magnetic and electric signals differ in their ability to facilitate distinguishing closely spaced neurons using only extracellular spike waveforms. Lastly, we demonstrate how the solenoidal nature of magnetic fields enables new approaches to neural morphology reconstruction using off-the-shelf algorithms.

## 2 Results

### 2.1 Complementary information from extracellular neural fields

**Fig 1:**
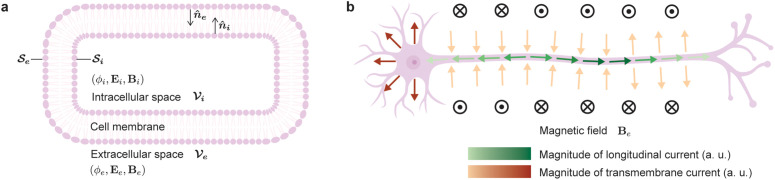
Application of Green’s theorem and neural currents diagram. **a**, Application of Green’s theorem to the intracellular space, ${𝒱}_{i}$, of a neuron—characterized by the potential $\phi_{i}$, electric field **E**_**i**_, and magnetic field **B**_**i**_, bounded by the surface ${𝒮}_{i}$ with local normal ${\hat{𝐧}}_{i}$—and to the extracellular space, ${𝒱}_{e}$—characterized by the potential $\phi_{e}$, electric field **E**_**e**_, and magnetic field **B**_**e**_, bounded by the surface ${𝒮}_{e}$ with local normal ${\hat{𝐧}}_{e}$. Membrane created in BioRender. Ali, **Z.** (2026) https://BioRender.com/fmjdzjs. **b**, Illustration of longitudinal and transmembrane currents generated by a neuron during an action potential, along with the direction of the extracellular magnetic field induced by longitudinal currents. Shading is normalized separately for the transmembrane and longitudinal currents to highlight their relative, rather than absolute, distributions. Cell created in BioRender. Ali, **Z.** (2026) https://BioRender.com/rbcfhct.

A neuron can be conceptually divided into intracellular, membrane, and extracellular regions (Fig 1a). Assuming nonlinear sources are confined to the membrane, the intra- and extracellular fields obey the Poisson equation, enabling use of Green’s theorem [28]. Previous work has applied this to express the extracellular potential in terms of both current densities and either the transmembrane potential or the inner and outer membrane potentials [29–31]. Here, we extend this approach using the vector form of Green’s theorem to relate the extracellular magnetic field to the current density on the membrane’s inner surface (see Supplementary Information, S1 Appendix for derivation).

For a source-free, extended, and non-magnetic extracellular space, the magnetic flux density generated by a neuron with intracellular electrical potential $\phi_{i}(𝐫)$ can be approximated as


$$\begin{array}{c} {𝐁}_{e}(𝐫)\approx \frac{\mu_{0}}{4\pi }\oint_{𝒮}\frac{-\sigma_{i}\nabla^{\prime }\phi_{i}({𝐫}^{\prime })\times {\hat{𝐧}}^{\prime }}{\left|𝐫-{𝐫}^{\prime }\right|}\;da^{\prime }, \end{array}$$ (1)

where $\hat{𝐧}$ is the normal to the membrane surface 𝒮 and $\sigma_{i}$ is the intracellular conductivity. Equation 1 is similar to the equation for the extracellular magnetic field found in [32] but is obtained without making any assumptions about the neuron’s geometry; the full expression in Supplementary Information Equation 9 (S1 Appendix) therefore includes additional terms absent from [32]. In comparison, the extracellular electrical potential is given by


$$\begin{array}{c} \phi_{e}(𝐫)\approx \frac{1}{4\pi \sigma_{e}}\oint_{𝒮}\frac{-\sigma_{i}\nabla^{\prime }\phi_{i}({𝐫}^{\prime })\cdot {\hat{𝐧}}^{\prime }}{\left|𝐫-{𝐫}^{\prime }\right|}\;da^{\prime }, \end{array}$$ (2)

where $\sigma_{e}$ is the extracellular conductivity. Therefore, the magnetic field depends on the tangential (longitudinal) component of the intracellular surface current density ${𝐢}_{is}^{l}=-\sigma_{i}\nabla \phi_{i}+(\sigma_{i}\nabla \phi_{i}\cdot \hat{𝐧})\;\hat{𝐧}$ on ${𝒮}_{i}$, while the electric potential depends on the normal (transmembrane) component, ${𝐢}_{is}^{t}=-(\sigma_{i}\nabla \phi_{i}\cdot \hat{𝐧})\;\hat{𝐧}$ on ${𝒮}_{i}$. Thus, as suggested in [33,34], magnetic and electric measurements capture complementary aspects of neural activity—currents flowing along and across the membrane, respectively (Fig 1b). We therefore hypothesize that simultaneous recordings of both modalities can provide a more complete picture of neuronal physical structure and a deeper understanding of neuronal dynamics across large populations.

### 2.2 Neural signal scaling

While the scaling of extracellular potentials generated by spiking neurons has been well studied [35–38], the distance-dependent behavior of extracellular magnetic fields remains relatively underexplored. Having established that extracellular magnetic fields and potentials arise from orthogonal components of the intracellular surface current density—${𝐢}_{is}^{l}$ (longitudinal) and ${𝐢}_{is}^{t}$ (transmembrane), respectively—we now examine how these components reverse sign along the neuron. Since, in the quasi-magnetostatic regime, both current sources generate fields with equivalent $\sim 1/R^{2}$ scaling, the spatial frequency of the sign changes of these currents determines the multipole order of the resulting fields, which in turn governs how the signal amplitude decays with distance (see Supplementary Information, S1 Appendix for detailed explanation).

To analyze the sign reversal in current components, we begin by computing the intracellular potential, from which both the longitudinal (${𝐢}_{is}^{l}$) and transmembrane (${𝐢}_{is}^{t}$) surface current densities are derived. We model the neuron as an infinitely long cylindrical axon of radius *a*, embedded in an unbounded volume conductor. Under these assumptions, the intracellular and extracellular potentials, $\phi_{i}(\rho ,z)$ and $\phi_{e}(\rho ,z)$, respectively, satisfy Laplace’s equation in cylindrical coordinates. The general solutions involve modified Bessel functions and are expressed as integrals over spatial frequency. The weighting terms for these Bessel components are determined by applying boundary conditions at the membrane.

To model the neuron’s activity during firing, we follow the approach in [39–41] assuming the transmembrane potential $\phi_{m}(z)$, which represents the action potential, is given. At the membrane (ρ=a), we impose the boundary condition $\phi_{i}(a,z)-\phi_{e}(a,z)=\phi_{m}(z)$, along with continuity of the normal component of current across the boundary. These constraints determine the weighting terms in the Bessel-function solutions. Since the axon is infinitely long, we perform the analysis in the Fourier domain, where axial variations simplify algebraically. Given that the length of the axon is much greater than its radius, we apply small-argument approximations to the Bessel functions to simplify the solution. This yields a tractable expression for the intracellular potential $\Phi_{i}(\rho ,k)$, from which the surface current components follow. Taking the inverse Fourier transforms, we obtain:


$$\begin{array}{c} i_{is,z}^{l}(z)\approx -\sigma_{i}\delta^{\prime }(z)*\phi_{m}(z) \end{array}$$ (3a)

$$\begin{array}{c} i_{is,\rho }^{t}(z)\approx \frac{\sigma_{i}a}{2}\delta^{\prime \prime }(z)*\phi_{m}(z). \end{array}$$ (3b)

where * denotes convolution and δ(·) is the Dirac delta function (see Supplementary Information, S1 Appendix for derivation).

When $\phi_{m}(z)$ is a unimodal function, such as a Gaussian, it does not change sign. Thus, the number of sign reversals in the current components is entirely determined by the order of differentiation. The longitudinal current $i_{is,z}^{l}(z)$, involving the first derivative of $\phi_{m}(z)$, reverses sign once, while the transmembrane current $i_{is,\rho }^{t}(z)$, involving the second derivative, reverses sign twice. In general, higher-order derivatives produce more sign changes, corresponding to higher-order multipoles, which decay more rapidly with distance. Specifically, the extracellular magnetic field—generated by the dipole-like structure of $i_{is,z}^{l}$—scales with distance *R* as 1/*R*^3^. In contrast, the extracellular electric field and potential, shaped by the quadrupolar structure of $i_{is,\rho }^{t}$, scale as 1/*R*^4^ and 1/*R*^3^, respectively, when referenced to a distant point. In practice, extracellular potentials are measured relative to a local reference electrode; if this reference is near the measurement electrode, the recorded potential approaches the electric field’s scaling behavior, 1/*R*^4^. As a result, the observed scaling typically falls between these two limits and can be approximated as $1/R^{3+\epsilon }$, where 0≤ϵ≤1, with ϵ reflecting the proximity of the reference electrode.

**Fig 2:**
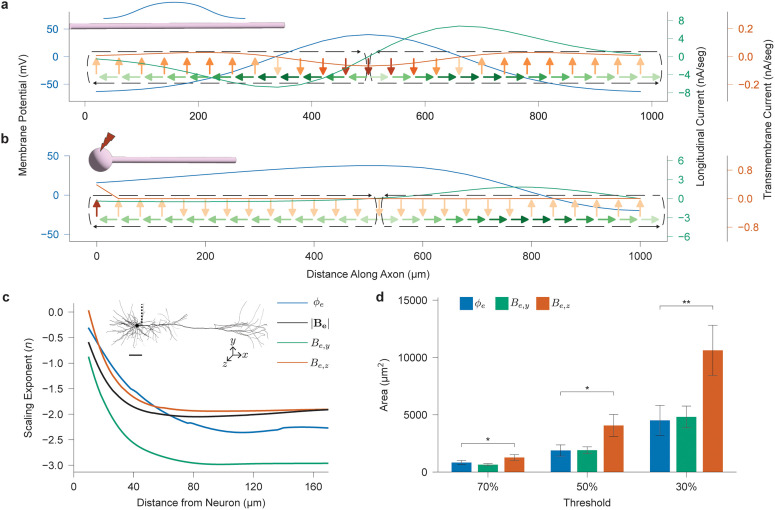
Scaling analysis of intracellular currents and extracellular fields in neuron models. **a**, Analytically-derived distribution of longitudinal $i_{is,z}^{l}$ and transmembrane $i_{is,\rho }^{t}$ components of the intracellular surface current along an infinitely extended cylindrical axon with a Gaussian-distributed membrane potential $\phi_{m}$, characterized by a standard deviation of 167 μm. The black dashed lines indicate current loops composed of the transmembrane and longitudinal currents which propagate down the length of the axon during the action potential. **b**, Cellular current distributions for a ball-stick cell model when the action potential peak travels half of the axon length. Parameters: axon length = 1 mm; axon diameter = 2 μm; soma diameter = 20 μm; segment length = 0.5 μm. **c**, Scaling exponent *n* characterizing how the extracellular potential $\phi_{e}$ and magnetic field **B**_**e**_, including the planar (*B*_*e*,*y*_) and normal (*B*_*e*,*z*_) components relative to the sensor array plane, vary as a function of distance from the cell (*R*^*n*^). The upper part of the figure shows the cell model (Cell 2 from S1 Fig) and measurement locations used to evaluate the scaling, marked by the black dashed line. Scale bar, 100 μm. **d**, Total area of supra-threshold signal for electrical potential $\phi_{e}$ and magnetic field components *B*_*e*,*y*_, *B*_*e*,*z*_, averaged for cells 0–5 (S1 Fig). Threshold is defined relative to the absolute value of the peak signal for each cell across the total time duration of a spike. Measurement point pitch set at 5 μm, so each location with a supra-threshold signal corresponds to an area of 25 $\mu {\text{m}}^{2}$. *p* = 0.042, 0.013, and 0.002 for thresholds 70%, 50%, and 30% respectively, computed using paired-samples t-test.

To validate and illustrate these theoretical predictions, we now proceed with three numerical evaluations. First, using a Gaussian function for $\phi_{m}(z)$ to represent an action potential, Fig 2a shows the distribution of $i_{is,z}^{l}(z)$ and $i_{is,\rho }^{t}(z)$ along a cylindrical axon, as derived from (3). These currents form two current loops, *n*, (black dashed arrows in Fig 2a), corresponding to traveling waves of depolarization, which indicate the number of sign flips for the longitudinal (*n*) and transmembrane (*n* + 1) current components. These results confirm the dipole behavior of an extended neural process’s magnetic field and the quadrupole behavior of its electrical potential during an action potential.

Next, we implement a ball-stick model to compute the corresponding transmembrane and longitudinal currents. Fig 2b shows the current distribution at the time when the action potential peaks at one-half of the length of the axon. Unlike a cylindrical axon, the presence of the soma significantly amplifies the transmembrane current at the proximal end relative to the rest of the axon. As a result, the quadrupole effect on the electric potential is reduced, suggesting that the scaling behavior for the extracellular electric potential may be better than $1/R^{3+\epsilon }$. Similarly, the forward longitudinal current in the axon is stronger than the reverse, indicating that the dipole effect on the magnetic field is diminished, and its scaling behavior may be better than 1/*R*^3^.

To evaluate how the non-uniform distribution of transmembrane and longitudinal currents in real cells skews electrical potential and magnetic field scaling away from the 1/*R*^3^ first-order approximation, we simulate six morphologically realistic layer V rat somatosensory cortical neurons (S1 Fig), all sourced from the Blue Brain Project [42]. These models were selected for their highly detailed morphology and biophysical realism, as well as their resemblance to the information-rich neurons commonly targeted in human studies. The set includes at least one representative of each pyramidal neuron type in the Blue Brain Project’s layer V repository (TTPC1, TTPC2, UTPC, STPC) and two interneuron types (DBC, MC). While all of the chosen cells are cortical neurons, the scaling trends in Equation 3 apply to all extended neural processes irrespective of cell type, including giant axons and peripheral nerves.

Fig 2c presents the scaling exponent *n* of the extracellular electrical potential $\phi_{e}$ and magnetic field **B**_*e*_ calculated along a radial line of measurement points extending outward from near the soma and adjacent to the apical dendrite of Cell 2 from S1 Fig during a spike (Fig 2c, inset). We also report the exponents for the planar, *B*_*e*,*y*_, and normal, *B*_*e*,*z*_, components of **B**_*e*_, as most magnetic sensors are sensitive to only one directional axis. The simulations reveal that the asymmetry in transmembrane and longitudinal current distributions leads to deviations from the expected 1/*R*^3^ scaling. This deviation is more pronounced for the magnetic field, which decays approximately as 1/*R*^2^ at large distance, compared to the electrical potential, which decays around 1/*R*^2.5^. Notably, the two magnetic field components exhibit distinct scaling behaviors: the planar component *B*_*e*,*y*_ decreases roughly an order of magnitude faster than the normal component *B*_*e*,*z*_. This discrepancy arises from the geometrical arrangement of the neuron relative to the measurement points. In conventional *in vitro* setups, neurons lie flat on top of probes composed of an array of sensors (e.g., Hall effect, magnetoresistive) that measure either *B*_*e*,*x*_, *B*_*e*,*y*_, or *B*_*e*,*z*_ fields. If the array is positioned a small distance Δz beneath the neuron, then for R≫Δz, the sensors are effectively coplanar with the neuron. In this geometry, the magnetic field for R≫Δz points primarily in the normal direction, as shown in S2 Fig, rendering the planar field components negligible and favoring arrays composed of *B*_*e*,*z*_ sensors. These asymptotic scaling trends are validated for the other morphological cell models in S3 Fig.

To understand the practical implications of these scaling trends for sensing, we evaluate, for each cell, the total area around the cell in the measurement plane where the signal magnitude exceeds some fraction of the overall maximum signal for $\phi_{e}$, *B*_*e*,*y*_, and *B*_*e*,*z*_. Fig 2d presents the results of this analysis for thresholds of 30%, 50%, and 70% of the peak signal. We observe that across thresholds, the total supra-threshold area for the *B*_*e*,*z*_ signal is approximately 2× that of the $\phi_{e}$ signal. This is due to the asymptotic scaling behavior illustrated in Fig 2c, but also to the distribution of longitudinal currents in the cell. Whereas the transmembrane current distribution is concentrated heavily in the soma, with weak return currents distributed across the entire rest of the cell (that do not generate strong signals), the propagation of the longitudinal currents down the major cellular processes, as shown in Fig 2a and 2b, generate a correspondingly widely distributed magnetic field signal. This can be seen in S1 Video as well as S4 Fig; while the electrical potential $\phi_{e}$ is largely radially symmetric around the soma, the *B*_*e*,*y*_ and *B*_*e*,*z*_ fields are more reflective of cellular morphology. This also explains why the *B*_*e*,*y*_ field, despite having worse radial scaling behavior than $\phi_{e}$, has approximately similar supra-threshold signal areas in Fig 2d, because the longitudinal current distribution offsets the poor radial scaling property. These results suggest that for *in vitro* measurements where sensor resolution is often limited, it may be more advantageous to measure normal magnetic fields than electrical potentials. This advantage has important implications for sensor design: targeting the normal field component can help optimize sensitivity and improve signal detectability, particularly in systems constrained by spatial sampling density or noise thresholds.

### 2.3 Cellular spatial resolution limit

Building on the complementary information and scaling analysis, we now turn to a core functional question: how distinguishable are neurons based on their extracellular magnetic fields compared to their electrical potentials? Distinguishability is fundamental to decoding neural activity from extracellular recordings; however, these recordings compress the rich, 3D spatial information generated by the unique morphological distribution of longitudinal and transmembrane currents for each neuron into a sparse, 2D *spike template* captured by a planar sensor array. This dimensionality reduction complicates the differentiation of neurons that are spatially proximate and/or morphologically similar.

We assess neuronal distinguishability across three levels of increasing complexity—pairwise cell discrimination, network-level separability, and spike sorting performance under realistic conditions. We hypothesize that magnetic field signatures may offer superior distinguishability due to three key differences: (1) magnetic fields decay more slowly with distance, potentially extending the spatial footprint of each neuron’s signal; (2) they are vectorial and sensitive to both position and orientation, unlike scalar electric potentials; and (3) they reflect longitudinal currents along axonal and dendritic branches, rather than being dominated by somatic transmembrane currents alone. To test this hypothesis, we begin by quantifying the fundamental spatial resolution limit: the minimum intercellular distance required to reliably distinguish two neurons based on their extracellular electric or magnetic signals.

We analyze the six previously described layer V neuron models by generating simulated spike templates for both the extracellular electrical potential, $\phi_{e}$, and the three Cartesian components of the magnetic field, *B*_*e*,*x*_, *B*_*e*,*y*_, and *B*_*e*,*z*_. For brevity, we refer to the extracellular potential as ϕ and the magnetic field components as *B*_*x*_, *B*_*y*_, and *B*_*z*_ henceforth. We define the *base templates*—which are spike templates in which the cell is located at the origin—as ${𝐌}_{n}^{s}$ for neuron *n* and signal $s\in \{\phi ,B_{x},B_{y},B_{z}\}$. These base templates are *N*_*R*_ × *N*_*T*_ matrices, where *N*_*R*_ denotes the number of spatial measurement points (sensors) and *N*_*T*_ is the number of time steps over the course of a single spike event. In our simulations, each neuron is positioned at the center of a dense grid of measurement points, with its major process oriented along the positive *x*-axis. The grid consists of 1,500 × 1,500 measurement points with a 2-μm pitch, effectively approximating a continuous measurement space with near infinitesimal spatial resolution. This setup allows us to isolate the effects of neuronal morphology and field patterns, independent of constraints imposed by real-world sensor spacing.

We investigate how overlapping spike signatures from two neurons, separated by a displacement Δ𝐫=(Δx,Δy), may appear similar due to spatial proximity and orientation. To simulate this effect, we generate new spike templates from the base template by applying spatial transformations: a rotation by angle θ and a translation by Δ𝐫 (S5 Fig). These transformed templates are denoted as $T_{trans}(\Delta 𝐫)\;T_{rot}(\theta )\;{𝐌}_{n}^{s}$, where $T_{rot}(\theta )$ and $T_{trans}(\Delta 𝐫)$ represent the respective rotation and translation operators. To quantify the minimum resolvable intercellular distance, we introduce a cosine similarity metric that measures the similarity between the spike signature of neuron *n* and that of a spatially transformed neuron *m* for signal *s*:


$$\begin{array}{c} R_{n,m}^{s}(\Delta 𝐫)=\frac{1}{2\pi }\int_{0}^{2\pi }\frac{{𝐌}_{n}^{s}\cdot T_{trans}(\Delta 𝐫)T_{rot}(\theta ){𝐌}_{m}^{s}}{\left\|{𝐌}_{n}^{s}\|\|T_{trans}(\Delta 𝐫)T_{rot}(\theta ){𝐌}_{m}^{s}\right\|}\;d\theta . \end{array}$$ (4)

By averaging over all rotation angles, we account for variations in neuronal orientation. A high value of $R_{n,m}^{s}(\Delta 𝐫)$, approaching 1, indicates that the spike signatures of neurons *n* and *m* are highly similar for signal type *s* at the displacement Δ𝐫, suggesting that the two neurons produce nearly indistinguishable extracellular fields and are therefore difficult to resolve. Conversely, a low or negative $R_{n,m}^{s}(\Delta 𝐫)$ signifies a high degree of dissimilarity between the spike signatures, indicating that the neurons can be more easily distinguished.

Under the assumption of infinitesimal pitch, the translation operator $T_{trans}(\Delta 𝐫)$ can be factored outside the integral, and the rotation integral interpreted as an orientation-averaging operator *T*_*sweep*_, yielding


$$\begin{array}{c} R_{n,m}^{s}(\Delta 𝐫)=\frac{{𝐌}_{n}^{s}\cdot T_{trans}(\Delta 𝐫)T_{sweep}{𝐌}_{m}^{s}}{\left\|{𝐌}_{n}^{s}\|\|T_{trans}(\Delta 𝐫){𝐌}_{m}^{s}\right\|}, \end{array}$$ (5)

where


$$\begin{array}{c} {𝐆}_{n}^{s}:=T_{sweep}{𝐌}_{n}^{s}=\frac{1}{2\pi }\int_{0}^{2\pi }T_{rot}(\theta ){𝐌}_{n}^{s}d\theta . \end{array}$$ (6)

**Fig 3:**
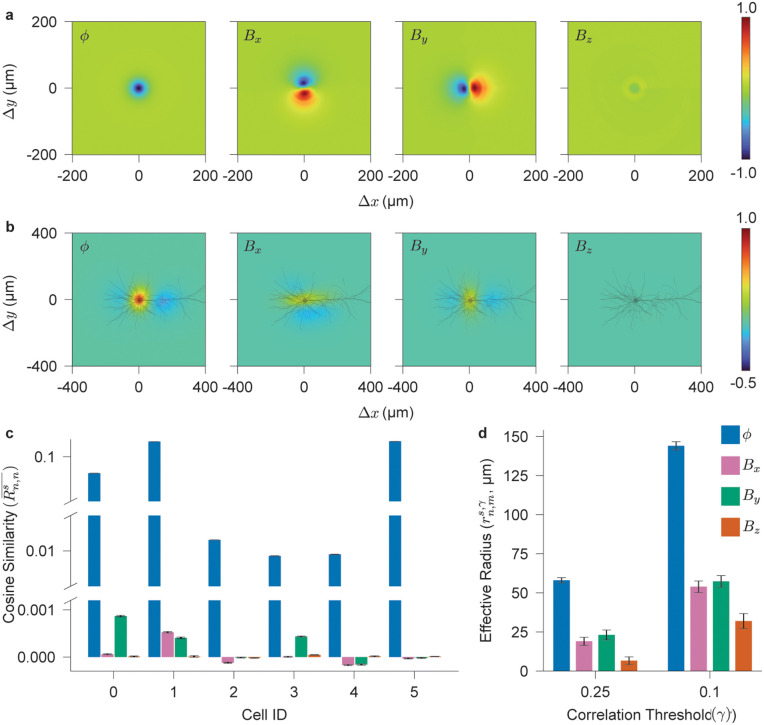
Spatial resolution limits of simulated neural extracellular electrical potentials and magnetic fields. **a**, Spread templates ${𝐆}_{n}^{s}$ for cell *n* = 2 and signals $s\in \{\phi ,B_{x},B_{y},B_{z}\}$. **b**, Spatial auto-similarity $R_{n,n}^{s}(\Delta 𝐫)$ for cell *n* = 2 and signals $s\in \{\phi ,B_{x},B_{y},B_{z}\}$. **c**, Mean spatial auto-similarity for Cells 0–5 from S1 Fig, averaged over displacements along both *x*- and *y*-axes within ±400 μm. **d**, Effective radius for high (γ>0.25) and moderate (γ>0.1) similarity thresholds, averaged over all cell pairs (*n*,*m*) from S1 Fig.

We refer to ${𝐆}_{n}^{s}$ as the *spread template*, as it characterizes how neuronal signals disperse through space irrespective of orientation, providing a basis for evaluating neuronal distinguishability based solely on spatial separation. Fig 3a displays the spread templates for Cell 2 at the time corresponding to the maximum overall template signal power. For the magnetic field, the rotation transformation is applied directly to the full vector field **B**_*e*_. Consequently, the vector-valued spread template is invariant to orientation. However, the individual components—specifically *B*_*x*_ and *B*_*y*_—are not, as they depend on how the rotated field projects onto their respective sensing axes. For visualization, the electrical potential template is normalized to its maximum value, while the magnetic field components are scaled by the maximum value across all three components to ensure consistent relative amplitudes. This normalization results in a lower relative magnitude for the *B*_*z*_ component compared to *B*_*x*_ and *B*_*y*_.

Fig 3b shows the auto-similarity, $R_{n,n}^{s}(\Delta 𝐫)$ for Cell 2, evaluated for each signal type $s\in \{\phi ,B_{x},B_{y},B_{z}\}$. This auto-similarity is essentially the convolution of ${𝐆}_{n}^{s}$ with ${𝐌}_{n}^{s}$ across the 2D space. The electrical potential exhibits high similarity with a radially symmetric distribution centered at the soma—consistent with the complementary information section, where we identified the soma as the primary source of transmembrane currents generating uniform-polarity extracellular signals. This symmetry is also evident in the spread template for ϕ (Fig 3a), indicating that neurons positioned around and near the soma produce highly similar extracellular potentials. In contrast, magnetic fields show lower spatial similarities. The planar magnetic fields *B*_*x*_ and *B*_*y*_ exhibit antisymmetric spread templates with respect to their sensing axes (Fig 3a), due to their sensitivity to neuronal orientation. For instance, rotating a neuron by 180° reverses the *B*_*y*_ signal, flipping the spread template across the *y*-axis. This leads to lower similarity and a triphasic spread template pattern slightly elongated along the sensing axis. The normal magnetic field (*B*_*z*_) is even more sensitive to rotation and translation. Sensors on opposite sides of the neuron’s extended structure detect signals of opposite polarity, as shown in Fig 1b, and even small rotations around the soma or translations perpendicular to the neuron’s major process can render the *B*_*z*_ template nearly anti-similar with the original. Consequently, the normal magnetic field results in minimal spatial similarity, substantially lowering the separation limit for distinguishing neurons—outperforming planar magnetic fields and, even more so, electrical potentials.

Fig 3c presents the spatial auto-similarity, averaged over displacements along both the *x* and *y* axes within a range of ±400 μm, for all six cells in S1 Fig. On average, electrical potential templates exhibit similarity values several orders of magnitude higher than those of magnetic field templates—even for cells *n* = 2, 3, 4, where extensive dendritic branching reduces radial symmetry. Planar magnetic field templates occasionally show non-negligible similarity or even anti-similarity, due to their antisymmetric spread templates and sensitivity to neuronal orientation. In contrast, normal magnetic field templates consistently exhibit near-zero similarity, making them particularly well-suited for recording and analyzing densely packed neuronal populations.

Finally, we quantify the spatial resolution limit—the minimum intercellular distance required for reliable discrimination—by defining an effective radius $r_{n,m}^{s,\gamma }$ as the radius of a circle with an area equal to the region where the similarity $R_{n,m}^{s}(\Delta 𝐫)$ exceeds a threshold γ:


$$\begin{array}{c} r_{n,m}^{s,\gamma }=\sqrt{|\{\Delta 𝐫:R_{n,m}^{s}(\Delta 𝐫)>\gamma \}|\cdot \frac{\Delta x\Delta y}{\pi }}. \end{array}$$ (7)

Here, |·| denotes the cardinality of the set. Fig 3d shows the effective radii averaged across all cell pairs (*n*, *m*) for two thresholds: 0.25 (high similarity) and 0.1 (moderate similarity). As expected, magnetic fields achieve higher spatial resolution than electrical potentials. Planar magnetic field templates yield effective radii approximately three times smaller than those of electrical potentials, while normal magnetic fields provide even greater resolution—up to five- to nine-fold improvements. These results indicate that magnetic sensors, especially those measuring the normal field, are fundamentally better suited for distinguishing neurons in densely populated regions than traditional electrical recordings.

### 2.4 Multi-source discrimination in practical sensor arrays

Having shown that magnetic fields offer superior pairwise discrimination under ideal spatial sampling, we now examine how this advantage scales to more realistic network-level settings. In particular, we investigate how sensor sparsity—an unavoidable constraint in practical arrays [43]—affects the ability to distinguish multiple simultaneously active neurons across space. Achieving broader coverage to monitor more neurons requires reducing sensor density, which in turn lowers spatial resolution and degrades the measurement quality for individual neurons. To evaluate how signal modality affects this trade-off, we adopt a metric from communication theory—the condition number—to quantify network-wide neuronal distinguishability. In addition to analyzing individual field components, we also examine combinations, such as $\left(\phi ,B_{z}\right)$, to assess whether integrating complementary electrical and magnetic recordings improves performance. In these multimodal configurations, each measurement location includes multiple sensors capable of recording different fields.

We use the condition number as a measure of signal separability, drawing from its established role in communication systems like massive MIMO and spread-spectrum CDMA [44,45]. In these systems, multiple overlapping signals are distinguished using non-orthogonal codes. If these codes are combined into a channel matrix, the condition number—defined as the ratio of the largest to smallest singular values of the channel matrix’s singular value decomposition—quantifies how well these signals can be separated. A value close to 1 indicates good separability, while a large value signals poor separation, especially in noisy environments. Similarly, if we create channel matrices consisting of the flattened spike templates of *N*_*C*_ randomly oriented and positioned neurons, we can compute the resultant condition number to assess how well different extracellular signal types support neuronal discrimination in crowded networks.

We use the condition number to evaluate neuronal distinguishability in two experimental scenarios. First, we fix the number of sensors and vary the sensor pitch—50, 75, and 100 μm—in a 10 × 10 array to examine how increasing the coverage area affects distinguishability, while maintaining a constant cell density of 340 cells/mm^2^ [46]. Second, we fix the sensor pitch at 75 μm—thereby holding the coverage area constant—and vary the number of neurons to assess how many cells an array can distinguish within a given condition number threshold.

**Fig 4:**
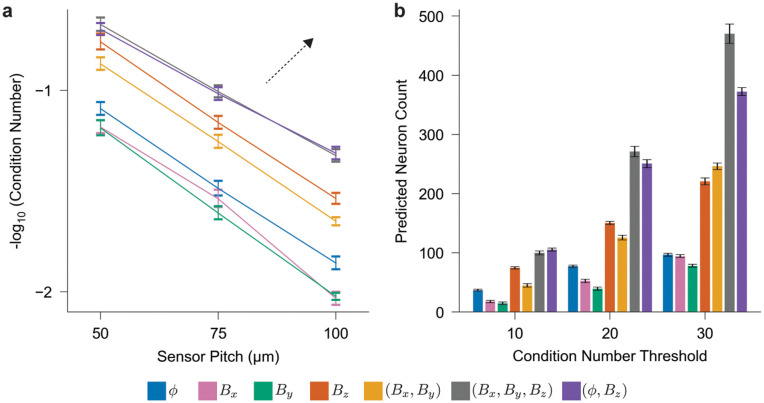
Multi-source discrimination analysis of neural electrical potential and magnetic field templates. **a**, Negative logarithm of the average condition number for channel matrices constructed from constant-density populations of *N*_*C*_ = 50, 113, and 200 cells (Cells 2–3 from S1 Fig) corresponding to sensor array pitch of 50, 75, and 100 μm, respectively, for signal types $s\in \{\phi ,B_{x}$, *B*_*y*_, *B*_*z*_, (*B*_*x*_, *B*_*y*_), (*B*_*x*_, *B*_*y*_, *B*_*z*_), (ϕ, *B*_*z*_)} (*N* = 30). The dashed arrow highlights the direction of a more favorable trade-off between neuronal distinguishability and coverage area. **b**, Maximum number of distinguishable neurons at various condition number thresholds, estimated via bootstrap resampling of the data shown in S6b Fig.

Fig 4a presents the trade-off between neuronal distinguishability—measured as the inverse of the condition number—and array coverage using channel matrices constructed from Cells 2 and 3 in S1 Fig. These neurons were chosen due to their strong, linear processes which can most clearly demonstrate the distinction between electrical and magnetic field information; an analysis of all cell types can be found in S6a Fig. Combining electrical potential and normal magnetic field measurements $\left(\phi ,B_{z}\right)$ achieves the optimal trade-off, supporting our hypothesis that these signals provide complementary information. The *B*_*z*_ array significantly outperforms the ϕ array, aligning with our pair-wise analysis, whereas the *B*_*x*_ and *B*_*y*_ arrays under-perform the ϕ array, contradicting that analysis. This discrepancy arises as an artifact of the *in vitro* measurement setup because neuronal processes lying flat above a plane of sensors and aligned parallel with the sensing axis generate magnetic fields orthogonal to it (via the right-hand rule), making such neurons nearly undetectable. Measuring both planar components (*B*_*x*_, *B*_*y*_) mitigates this orientation sensitivity and yields performance comparable to the *B*_*z*_-only array, which is inherently less susceptible to orientation-dependent signal loss on a planar array. Simultaneous measurement of all three magnetic components (*B*_*x*_, *B*_*y*_, *B*_*z*_) captures additional information and further improves performance, closely approaching that of the hybrid $\left(\phi ,B_{z}\right)$ array.

Fig 4b presents results for the second experimental scenario, where the sensor pitch is fixed at 75 μm and 20–200 neurons are sampled from Cells 2 and 3 from S1 Fig to assess the effect of increasing cell density. For each array type, we use bootstrapping of the data in S6b Fig to evaluate the maximum number of cells each array can distinguish at the same distinguishability, or condition number, threshold. At nearly all tested thresholds, the *B*_*z*_ array supports approximately twice as many neurons as the ϕ array, while the $\left(\phi ,B_{z}\right)$ and (*B*_*x*_, *B*_*y*_, *B*_*z*_) arrays support up to three to four times more neurons. However, these multimodal arrays require two to three times as many sensors as the conventional ϕ-only configuration. In contrast, the *B*_*z*_ array outperforms the ϕ array even when constrained to the same number of sensors. These findings suggest that *B*_*z*_ arrays provide an efficient and scalable solution for large-scale neural recordings, particularly under practical constraints such as limited data bandwidth and sensor fabrication complexity.

### 2.5 Spike sorting

Building on our previous findings—namely, that dense populations of cortical neurons are more readily distinguishable via their extracellular magnetic fields than their electrical potentials, and that combining complementary electrical and magnetic recordings further enhances separability—we now investigate how these metric-based differences translate to performance differences in realistic scenarios using *in silico* spike sorting. We simulate spike sorting using both *in vitro* and *in vivo* array configurations. The *in vitro* arrays are square, planar sensor arrays with varying pitches (S7a Fig) whereas the *in vivo* array replicates the configuration of a Neuropixel 1.0 probe [6] (S7b Fig). This setup allows us to compare sorting performance across different spatial layouts and experimental conditions. Simulated recordings are generated by randomly distributing a population of neurons across the arrays, summing and concatenating their Poisson-generated spikes, and subsequently adding noise. Spike sorting is performed using the Kilosort2 algorithm [47]. The full sorting pipeline is shown in S8 Fig. Performance is evaluated using two metrics: overall sorting accuracy, defined as the match rate between predicted and ground truth spike trains, and the number of well-detected cells, defined as units with match rates exceeding 80% [48]. These well-detected cells, which typically correspond to one-to-one matches with ground truth neurons, reduce the need for manual curation, such as merging or splitting spike templates [49].

**Fig 5:**
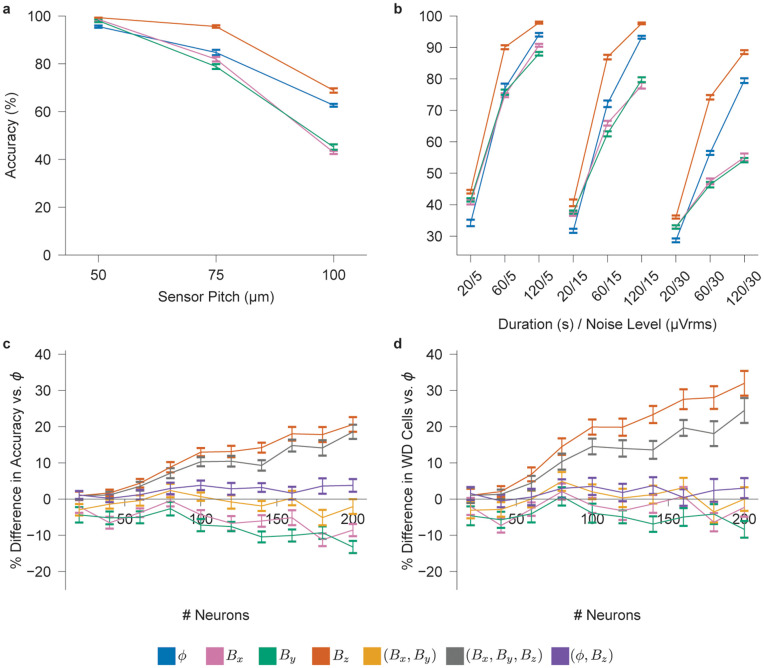
Simulated spike sorting for *in vitro* neural probes. **a**, Spike sorting accuracy for $s\in \{\phi ,B_{x},B_{y},B_{z}\}$ as a function of sensor pitch (50, 75, and 100 μm; corresponding to 50, 113, 200 cells at constant density; 60 s duration, 15 $\mu {\text{V}}_{rms}$ noise level). The noise level for magnetic templates is in units of tesla and is normalized such that their average SNR matches that of the ϕ template. **b**, Sorting accuracy as a function of recording duration (20, 60, 120 s) and noise level (5, 15, 30 $\mu {\text{V}}_{rms}$) for each signal type (200 cells, 75 μm pitch). **c**, Percent difference in spike sorting accuracy relative to ϕ for signal types $s\in \{B_{x},B_{y},B_{z},(B_{x},B_{y}),$ $\left(B_{x},B_{y},B_{z}),(\phi ,B_{z})\right\}$ as a function of cell count (75 μm pitch, 60 s duration, 15 $\mu {\text{V}}_{rms}$ noise level). **d**, Percent difference in the number of well-detected cells (match rate >80%) under the same conditions as in **c**. *N* = 10 for all panels.

Fig 5a presents the trade-off between spike sorting accuracy and coverage area for *in vitro* arrays with a fixed number of sensors. Across all coverage areas, the normal magnetic field *B*_*z*_ consistently yields higher sorting accuracy than the electrical potential ϕ, while the planar magnetic components *B*_*x*_ and *B*_*y*_ perform worse than ϕ except at the smallest coverage area. To further examine these trends, we evaluate how recording duration and noise level affect sorting performance. As shown in Fig 5b, the relative ranking of spike sorting accuracy for all signal types remains consistent as a function of these variations. As expected, accuracy improves with longer recording durations and degrades with increased noise across all signal types. While the performance of ϕ and *B*_*z*_ declines gradually with increasing noise, the degradation in *B*_*x*_ and *B*_*y*_ is significantly steeper, reflecting the same pronounced sensitivity to coverage area (Fig 5a).

These findings align with our earlier multi-source discrimination analysis, in which *B*_*x*_ and *B*_*y*_ exhibited significantly larger condition numbers—indicating poor signal separability—while the normal magnetic field *B*_*z*_ is consistently better conditioned. Although noise was not explicitly considered in that analysis, the sharper decline in sorting accuracy for *B*_*x*_ and *B*_*y*_ under noise reinforces their ill-conditioned nature. This demonstrates that although poorly conditioned channels are more susceptible to noise, their performance can be improved with sufficiently small sensor pitch. Overall, these findings support the condition number as a strong predictor of spike sorting algorithms’ ability to reliability separate and identify neural signals in practical settings.

Fig 5c evaluates how increasing cell density within a fixed coverage area affects sorting accuracy for *in vitro* arrays. To facilitate comparison, we plot the percentage difference in spike sorting accuracy between each signal type and the ϕ array. At low cell densities, the *B*_*z*_ and (*B*_*x*_, *B*_*y*_, *B*_*z*_) arrays perform comparably to the ϕ array; however, at the highest evaluated density, they yield nearly a 20% increase in sorting accuracy. In contrast, the *B*_*x*_ and *B*_*y*_ arrays consistently underperform the ϕ array, while the $\left(B_{x},B_{y}\right)$ and $\left(\phi ,B_{z}\right)$ arrays remain comparable to it. A similar trend appears in Fig 5d, where the *B*_*z*_ and (*B*_*x*_, *B*_*y*_, *B*_*z*_) arrays yield 20–30% more well-detected cells than the ϕ array, while other multimodal arrays show marginal or no improvement.

These results reaffirm the effectiveness of measuring the normal magnetic field component *B*_*z*_, alone or in combination with other fields, which is consistent with our distinguishability analysis identifying *B*_*z*_ as best suited for recording from dense neural networks. However, multimodal arrays—particularly $\left(\phi ,B_{z}\right)$—underperform relative to predictions from the distinguishability analysis. This discrepancy likely reflects limitations of the spike sorting algorithm rather than the signal content itself. Since Kilosort incorporates sensor position into its model, it may struggle with co-located heterogeneous signals. Additionally, the two- to three-fold increase in sensor count for multimodal arrays may further challenge algorithmic performance.

**Fig 6:**
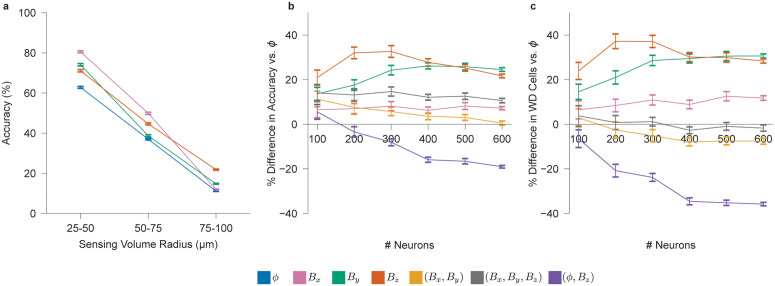
Simulated spike sorting for *in vivo* neural probes. **a**, Spike sorting accuracy as a function of distance from the probe for $s\in \{\phi ,B_{x},B_{y},B_{z}\}$ (400 cells, 120 s duration, 15 $\mu {\text{V}}_{rms}$ noise level). **b**, Percent difference in sorting accuracy relative to ϕ for signal types $s\in \{B_{x},B_{y},B_{z},(B_{x},B_{y}),(B_{x},B_{y},B_{z}),(\phi ,B_{z})\}$, as a function of cell count (120 s duration, 15 $\mu {\text{V}}_{rms}$ noise level, 10–100 μm cell radius). **c**, Percent difference in the number of well-detected cells under the same conditions as **b**. *N* = 10 for all panels.

Next, we turn to *in vivo* settings, where probes sample from a three-dimensional (3D) volume (S7b Fig), and only neurons near the probe are reliably detected, while distant cells contribute background noise [50]. Because sorting accuracy depends on the number of detectable neurons, we define an effective sensing radius—the distance beyond which neurons are excluded and treated as noise—and evaluate sorting accuracy across radial distances (S7c Fig). Fig 6a shows that the sorting accuracy for individual components (ϕ, *B*_*x*_, *B*_*y*_, *B*_*z*_) decreases linearly with radial distance, dropping below 25% between 75 and 100 μm. We therefore define 100 μm as the effective sensing radius, using 25% as a conservative threshold below which spike assignments are considered unreliable. To determine whether neurons beyond this radius should be treated as noise, we vary their number within a probe distance between 100–200 μm. Sorting accuracy remain largely unaffected across signal types, supporting the exclusion of biological noise from further simulations (S9 Fig).

Unlike the *in vitro* case, where we examine trade-offs between sorting accuracy and both coverage and cell density, the *in vivo* analysis focuses solely on the trade-off with cell density, as coverage is fixed by the probe geometry. As shown in Fig 6b, individual magnetic field arrays achieve up to 25–35% higher sorting accuracy than the ϕ array, even at the highest evaluated cell densities. In contrast, multimodal arrays underperform relative to the individual components, likely due to the same algorithmic limitations observed *in vitro*. While the (*B*_*x*_, *B*_*y*_, *B*_*z*_) array provides a modest 10% improvement over ϕ, the $\left(\phi ,B_{z}\right)$ array performs significantly worse. Fig 6c shows that well-detected cell counts follow similar trends.

Additionally, while the normal magnetic field *B*_*z*_ continues to outperform ϕ as in the *in vitro* case, it is no longer a clear outlier relative to the planar components. This shift likely stems from the three-dimensional cellular arrangement *in vivo*, where a neuron’s magnetic field may be oriented either normal or tangential to the probe surface—unlike the *in vitro* setting, where neurons predominantly lie in a plane orthogonal to the *B*_*z*_ sensing axis.

Together, these results demonstrate that the theoretical advantages of magnetic field sensing for neuronal discrimination extend to practical spike sorting scenarios, even when using algorithms originally developed for electrical potential recordings. Across both *in vitro* and *in vivo* settings, the normal magnetic field component *B*_*z*_ consistently enhances sorting accuracy and increases the number of reliably identified neurons, particularly in dense neural populations. While performance gains are partially limited by algorithmic assumptions–especially in multimodal, co-located configurations—the robustness of *B*_*z*_ across spatial scales and noise conditions highlights its value for next-generation neural interfaces. Future improvements may be realized through dedicated algorithmic development tailored to the unique characteristics of vectorial and composite magnetic recordings.

### 2.6 Morphological reconstruction

Thus far, we have examined how fundamental differences between neural electrical potentials and magnetic fields affect the separability of cells within large populations. We now shift focus to the single-cell level, exploring how the distinctive properties of neural magnetic fields can also inform the structural characterization of individual neurons. In particular, we explore *in vitro* morphology reconstruction, the task of estimating the spatial configuration of a neuron and its major processes. Traditionally, this task requires dense electrode arrays with inter-electrode spacing below 20 μm, along with complex algorithms or antidromic stimulation, due to the small radius and weak extracellular potential signal of neuronal processes relative to the soma [51–53]. Here, we investigate whether magnetic field measurements can facilitate morphology reconstruction using sparser arrays, which are more compatible with large-scale network recordings.

**Fig 7:**
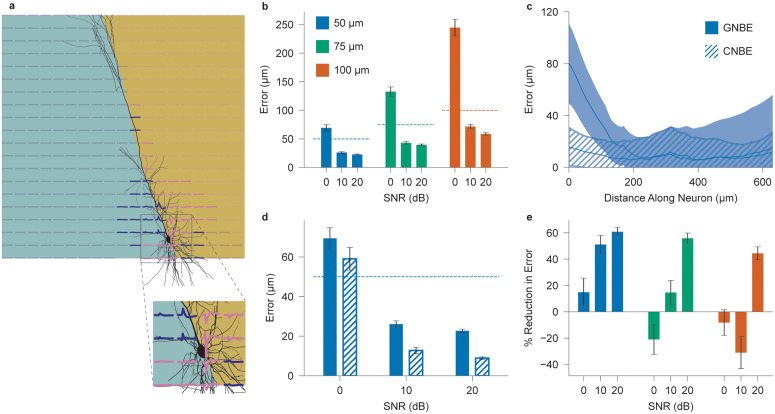
Morphology reconstruction for simulated *B*_*z*_ *in vitro* sensor arrays. **a**, Example of a decision boundary computed using the GNBE method, overlaid on the ground-truth neuron morphology. Spike signals at each sensor are shown, with positive signals in indigo and negative in pink. Inset shows how magnetic field polarities differ on opposite sides of the soma (top versus bottom) due to opposing longitudinal current flows. **b**, Reconstruction error using the GNBE algorithm for *B*_*z*_ templates with 0, 10, and 20 dB average SNR, across 20 × 20 sensor arrays with 50, 75, and 100 μm pitch. Dashed lines indicate corresponding values of sensor pitch. **c**, Average reconstruction error as a function of distance from the soma along the apical dendrite, comparing GNBE and CNBE methods for 20 dB-SNR, 50 μm pitch sensor array. **d**, Reconstruction error versus SNR for a 20 × 20 *B*_*z*_ array with 50 μm pitch, comparing GNBE and CNBE methods. **e**, Percent reduction in reconstruction error achieved by CNBE method under the same conditions as in **a**. *N* = 300 randomly oriented instances of Cell 2 from S1 Fig. Standard error is plotted for **b, d, e**, and standard deviation is shown for **c**.

As shown in Fig 1b, a planar *in vitro* array measuring normal magnetic fields from a closely positioned cell shows opposite polarities on either side of a major process, allowing its localization by detecting the boundary between positive and negative versions of the extracellular spike waveform. To demonstrate the feasibility with which this boundary can be estimated, we repurpose support vector machines (SVMs) for single-shot estimation—deviating from their conventional use in classification—and refer to this approach as General Neural Boundary Estimation (GNBE). SVMs are well-suited due to their off-the-shelf availability, training-free use in this context, and ability to compute contiguous, non-linear hyperplanes that better match the geometry of realistic neural processes compared to alternatives such as logistic regression or random forests [54]. In GNBE, a noisy *B*_*z*_ spike template—representing a single neuron’s estimated signal after spiking sorting—is input into the SVM (actual spike-sorted templates are not used so that the added noise in the template can be precisely controlled). Using sensor locations and signal polarities, the SVM computes a boundary curve interpreted as the dominant process’s orientation. This estimated boundary is then compared to the ground-truth morphology, as illustrated in Fig 7a. To assess feasibility, we simulate neurons on planar sensor arrays across varying sensor pitches and signal-to-noise ratios (SNRs), reflecting different levels of template quality.

Fig 7b shows the reconstruction error, defined as the average distance between each neuronal segment and the predicted boundary curve, as a function of template SNR and sensor pitch. For moderate (10 dB) and high (20 dB) SNRs, the reconstruction error is smaller than the sensor pitch for all pitch values tested, demonstrating super-resolution accuracy. At low SNR (0 dB), performance degrades considerably but improves with decreasing senor pitch.

While off-the-shelf GNBE-based reconstructions can attain high accuracy, they also exhibit consistent inaccuracies near the soma due to branching dendrites connected to the soma and oriented away from the primary neural process generating magnetic fields that rotate in the opposite direction (Fig 7a inset). To address this, we introduce a second-pass algorithm, referred to as Customized Neural Boundary Estimation (CNBE), that leverages known properties of cortical magnetic fields. CNBE proceeds in four steps: apply GNBE to obtain an initial boundary estimate; use this estimate to approximate the soma location and the direction of the major process; discard sensors on the side opposite the major process—typically associated with dendritic fields (Fig 7a, insert)—and re-estimate the boundary using only the remaining sensors. Full algorithmic details are provided in the Methods section.

Fig 7c shows the reconstruction error along the major process as a function of distance from the soma, comparing GNBE and CNBE algorithms for a 50 μm-pitch array. CNBE effectively eliminates the elevated error near the soma observed with the naive method. Fig 7d summarizes reconstruction error across all SNR levels for both algorithms. With CNBE, the error is reduced to approximately 20% of the pitch at moderate and high SNR, with modest improvement even at low SNR. Fig 7e quantifies the percentage reduction in reconstruction error achieved by the CNBE algorithm across all sensor pitches.

These results demonstrate that approximate morphology reconstruction at the cellular level, even using off-the-shelf tools as in GNBE, is enabled by the unique solenoidal properties of perpendicular neural magnetic fields. Furthermore, tailoring these tools to the known geometrical properties of neurons, as in the CNBE approach, yields significant improvement for dense arrays or high-SNR templates. While CNBE performs poorly on sparse arrays or low-SNR templates, as it then amplifies rather than corrects GNBE errors, it highlights the potential for more complex, custom algorithms to achieve even greater reconstruction accuracy. Neither GNBE nor CNBE are exhaustive — in particular, they are unlikely to work well for cells without major longitudinal processes, such as stellate cells or anaxonic neurons, as there is no single major boundary to estimate. However, their efficacy in this limited example provides an intuitive demonstration of how the unique informational content of perpendicular neural magnetic fields may facilitate new techniques.

## 3 Discussion

Extracellular magnetic fields and electrical potentials originate from distinct components of the intracellular surface current density—specifically, longitudinal and transmembrane currents, respectively. This biophysical distinction suggests that magnetic fields are particularly sensitive to extended neuronal structures such as dendrites and axons, whereas electrical potentials are dominated by somatic activity. As a result, magnetic recordings, especially from the normal component of the field, can provide richer spatial and morphological information for certain cell types. The rotational nature of magnetic fields also leads to more spatially diverse signal patterns, which may aid in resolving overlapping sources within densely packed neuronal populations. These insights motivate the integration of electrical and magnetic recordings to enhance both the resolution and interpretation of extracellular measurements.

To characterize differences between the two signal types, we first examined how electrical potentials and magnetic fields generated by spiking neurons scale with distance from their sources. While the electric field from transmembrane currents decays rapidly due to its quadrupole-like structure, the magnetic field—arising from longitudinal currents—exhibits dipole-like behavior with a slower decay. Extracellular return longitudinal currents were omitted from this analysis as they contribute negligibly to the magnetic field for cells in extended conductive volumes where the cell–sensor distance is less than the depolarization length of the cell [33], but they are an interesting topic for future study. Simulations using realistic morphologies showed that the normal magnetic field decays roughly as 1/*R*^2^, compared to 1/*R*^2.5^ for the electrical potential. This difference in spatial reach suggests that the normal magnetic field may retain more information at greater distances, offering advantages for recording in larger volumes and sparser sensor configurations.

Building on this foundation, we quantify how these signal properties influence the ability to distinguish individual neurons. We evaluate pairwise neuronal resolution using a spatial similarity metric applied to spike templates from morphologically realistic cells. While electrical potentials produce highly symmetric and correlated templates due to their somatic origin, magnetic fields—particularly, the normal magnetic field—capture more complex spatial features. This results in significantly improved resolution, with the normal magnetic field reducing the minimum resolvable pairwise separation distance by up to nine-fold compared to the electrical potential. Planar components also improve resolution but are more sensitive to neuronal orientation. This enhancement in resolution is calculated without considering the effects of tissue dielectric properties—which would further diminish the quality of electrical potential recordings—demonstrating that the difference in informational utility is intrinsic to neural magnetic fields. These results underscore the fundamental advantage of measuring normal magnetic fields for distinguishing neurons in dense networks and establish a strong basis for extending the analysis to larger populations.

To assess cell distinguishability at the network level, we apply the condition number—a communication theory metric for quantifying signal separability—to simulated spike templates of cortical populations. Across varying array coverages and cell densities, normal magnetic field templates are consistently better-conditioned than electrical potential templates, especially in populations with elongated neuronal processes. Planar magnetic components are poorly-conditioned due to orientation-dependent blind spots, where signals align orthogonally to the sensor axes. Although the advantage of normal magnetic sensing diminishes in populations lacking strong longitudinal structures, combining magnetic and electrical signals reliably improves separability, underscoring their complementary strengths. While multimodal configurations yield the highest gains in conditioning, they require substantially more sensors. Notably, sensing the normal magnetic field alone provides a strong trade-off, exceeding the performance of electrical potentials even under matched hardware constraints. These results underscore the practical utility of magnetic recordings for dense neural monitoring and support their scalability in settings limited by data throughput and fabrication complexity

We next evaluate whether the theoretical advantages of magnetic field sensing translate to practical spike sorting performance using simulated recordings processed with a sorting algorithm. Across both *in vitro* and *in vivo* configurations, recordings of the normal magnetic field consistently improve spike sorting accuracy and increase the number of well-detected units, especially in dense neuronal populations. Planar magnetic components perform less reliably in the *in vitro* setting but match the performance of the normal magnetic field *in vivo*, likely due to the three-dimensional organization of neural tissue, smaller sensor pitch, and greater sensitivity to distant sources. Multimodal recordings that combine electrical potential and magnetic field signals fall short of theoretical expectations, likely due to current algorithmic limitations in handling co-located, heterogeneous inputs. Nonetheless, spike sorting based on the normal magnetic field remains robust to noise, recording duration, and population density, suggesting that targeted algorithmic improvements could further enhance its utility.

Beyond improving conventional neural analyses, we explore how the unique characteristics of magnetic field measurements—particularly, the solenoidal nature of the normal component—can support structural inference at the single-cell level. Because this component exhibits polarity reversals across extended neural processes, we leverage this spatial pattern to estimate the orientation of major morphological structures. Using support vector machines (SVMs) repurposed for polarity-based boundary estimation, we achieve super-resolution accuracy even with low-SNR spike templates. A second-pass refinement, which excludes signals likely associated with dendritic polarity inversion near the soma, further improves performance. Under favorable conditions, this approach reduces reconstruction error by more than 50%. These results suggest that recordings of the normal magnetic field, even from sparse sensor arrays, can enable coarse morphology estimation in *in vitro* settings and offer structural insights that complement traditional spike-based analyses.

Our results point to a critical and underexplored direction for future sensor development: the need for highly sensitive magnetic sensors that can detect the normal component of neural magnetic fields. Currently, no magnetic sensor technology offers the necessary combination of sensitivity, size, and low noise to enable single-shot, single-cell recordings of cortical or peripheral activity. Consequently, experimental studies rely on extensive averaging, such as in [27], which combined thousands of recordings obtained with giant magnetoresistive (GMR) sensors to observe single-cell extracellular magnetic spikes. While averaging can help validate computational predictions — e.g., the measured amplitude of the neural magnetic fields in [27] is comparable to, although slightly larger, than we expect from our model — it precludes real-time sensing and introduces possible artifacts.

Magnetic tunnel junction (MTJ) sensors are one promising candidate for single-shot sensing. Due to their more common usage in electronic memory, they exhibit sub-micron size as well as compatibility with CMOS fabrication [24,26]; therefore, they can plausibly be directly integrated with processing circuitry and fabricated in large arrays to trade-off spatial resolution for enhanced detectivity. In addition, electrode contacts can be directly sputtered onto MTJ sensor wafers after microfabrication to integrate the two sensing modalities (as was attempted in [27] with a GMR probe). However, even the most sensitive MTJs are expected to generate only tens to hundreds of nanovolts in response to an action potential on the order of hundreds of picotesla, which is both below the intrinsic noise floor of most MTJs and prohibitively small to be detected by subsequent analog processing circuits. In addition, most MTJs are optimized for detecting planar fields; perpendicular MTJs remain far less explored and developed and, when fabricated, typically exhibit reduced sensitivity [55]. Single-shot, cellular-level magnetic field measurements will be contingent on improving the intrinsic sensitivity and noise performance of perpendicular MTJ sensors as well as developing methods to extrinsically enhance detectivity without significantly sacrificing spatial resolution. One method to accomplish this is to better leverage the vertical dimension when fabricating these sensors to facilitate denser packing, such as via the use of vertical flux concentrators [56]. Another major challenge will be to overcome the low-frequency flicker noise inherent to MTJs; this will require a chopping scheme, such as the one demonstrated in [57], but more miniaturizable and compatible with perpendicular sensors. Although overcoming the design challenges will require significant effort, our findings make clear that neural magnetic field sensing could dramatically enhance the capabilities and impact of next-generation implanted neural interfaces.

## 4 Methods

### 4.1 Neuron simulation and extracellular calculations

To calculate neural action potentials we used the NEURON simulator library [58], and to calculate extracellular electrical potentials we used the line-source approximation [59] within the LFPy module [60]. Spikes were elicited by injecting current into the soma using NEURON’s IClamp point process. The IClamp stimulus was weighted to generate a burst of spikes, and the first spike was omitted from all further analyses to eliminate artifacts from the stimulus itself. Current dipoles were computed for all cells as a sanity check using the method in [61] and compared to the values from that work. We obtained dipoles between 0.1–0.9 pA m, which is in agreement with the literature.

We used the MEArec package [62] to generate simulated extracellular electrical potential recordings from large populations and modified it to calculate and incorporate extracellular magnetic field data. Reference electrodes were assumed to be located at infinity. Magnetic field calculations were made using the Biot-Savart Law approximation [63] updated for a neuron model as shown in Eqn. 8, which describes the magnetic field at an extracellular coordinate **r** in terms of the neuron compartmental axial current *I*_*a*_, direction vector **d**, and relative position vector ${𝐫}^{\prime }$ (pointing from the compartment to the calculation point) for each compartment *n*.


$$\begin{array}{c} 𝐁(𝐫)=\frac{\mu_{0}}{4\pi }\sum_{n}I_{a,n}\frac{{𝐝}_{n}\times {\hat{𝐫}}_{n}^{\prime }}{\|{𝐫}^{\prime }{\|}^{2}} \end{array}$$ (8)

For both *in vitro* and *in vivo* arrays, cell morphologies were modified so as to neither intersect the plane of the array nor extend, unrealistically, upwards into space. For *in vitro* arrays, all cells were flattened to within a z-dimension of 5–25 μm above the plane of the sensors. For the *in vivo* arrays, all cellular processes that would have intersected the shank were made to instead wrap around the nearest shank boundary. Cells were also flattened so as to not cross within 5 μm of the front-face of the shank.

### 4.2 Similarity calculation and template creation

Simulated cells were suspended 15 μm (soma as the origin) over a 1,500×1,500 grid of measurement points with 2 μm pitch and flattened. This setup was intended to model a traditional *in vitro* electrophysiology experiment but with near-infinitesimal resolution. The signal traces of each sensor for the spike duration (224 timesteps, *dt* = 31.25 μs, 7 ms total) were combined to form a spike template ${𝐌}_{n}^{s}$ for each neuron *n* and signal type *s* (2,250,000×224, *n* = 0–5, $s\in \{\phi ,B_{x},B_{y},B_{z}\}$). To create ${𝐆}_{n}^{s}$, we defined the *T*_*sweep*_ transformation over θ from 0 to 360° in 15° increments. Translation coordinates Δ𝐫 for $T_{trans}(\Delta 𝐫)$ were defined over a coarse grid from (-400 μm, -400 μm) to (400 μm, 400 μm) with 50 μm pitch and a fine grid from (-200 μm, -200 μm) to (200 μm, 200 μm) with 20 μm pitch (in the *x*-*y* plane).

In generating templates for rotated cells, we had to contend with the fact that rotated sensor coordinates no longer necessarily aligned with the original sensor coordinates. Therefore, after obtaining the rotated coordinates, the *K*-nearest rotated sensors (*K* = 5) of each original sensor were determined. Then, the new template value $p_{0,\theta }$ at each original sensor location was calculated as the average of the data at the *K*-nearest neighbors inversely weighted by distance as described in (9) (for data *p*_*k*_ at the *K*-nearest neighbors with distance *d*_*k*_ and weights *w*_*k*_).


$$\begin{array}{c} w_{k}=\frac{\frac{1}{d_{k}}}{\sum_{k^{\prime }=1}^{5}\frac{1}{d_{k^{\prime }}}} \end{array}$$ (9a)

$$\begin{array}{c} p_{0,\theta }=\frac{\sum_{k=1}^{5}w_{k}p_{k}}{K} \end{array}$$ (9b)

### 4.3 Spike sorting recording generation

To generate the simulated recording data for spike sorting, we first created template libraries in which a sensor configuration was chosen (such as a 10 × 10 planar grid of sensors with 50, 75, or 100 μm pitch, or a Neuropixel 1.0 probe), a cell type was specified (Cell 2 or 3 from S1 Fig), and then *n* different spike templates of signal *s*—for $s\in \{\phi ,B_{x},B_{y},B_{z}\}$—for that cell were generated by randomly varying the location and rotation angle of the cell. For the Neuropixel, cells were placed only within some radius in front of the probe (volume defined by a half-cylinder) due to the inability of our simulation environment to adequately model transmembrane currents originating behind the probe (which would theoretically travel around it to reach microelectrodes rather than directly through it). All cells were flattened and/or wrapped around the sensor array.

Following the generation of a template library for each cell for a specified sensor configuration, *N*_*C*_ cell templates were randomly sampled across the combined templates of all selected cell types to represent the *N*_*C*_ neurons being simultaneously measured by the probe. A Poisson spike train was generated using MEArec for each template with a rate chosen from a normal distribution (mean = 5 Hz, standard deviation = 1 Hz) and the resultant extracellular signals were then combined to produce a recording. Spikes violating a set refractory period of 2 ms were removed.

Gaussian thermal noise was generated and added to voltage recordings according to a set noise power value. The range of noise powers used was chosen based on fundamental thermal noise properties of resistors. A typical impedance value (at 1 kHz) for an electrode with a size on the order of 10 μm is generally between 100 kOhm to over 1 MOhm depending on the electrode material [6,64,65]. The power spectral density of thermal noise from a resistor is 4*kTR*, so for an electrode measuring a signal in a 32 kHz bandwidth (equivalent to our *dt* for spike generation; data was band-pass filtered during spike sorting), this impedance range approximately corresponds to a noise RMS voltage range of 7.35 to 32.9 $\mu {\text{V}}_{rms}$. We chose to simulate from 5 to 30 $\mu {\text{V}}_{rms}$. While realistic electrode impedances scale as a function of frequency, assuming a constant resistance provides us with a simple and conservative estimate. Notably, the Neuropixel 1.0 probe reports approximately 7.1 $\mu {\text{V}}_{rms}$ noise for an *in vivo* implant within a 300 Hz to 10 kHz bandwidth [66]—this corresponds to 12.9 $\mu {\text{V}}_{rms}$ when the bandwidth is changed to 32 kHz. We therefore chose 15 $\mu {\text{V}}_{rms}$ as our default noise level for the *in vitro* and *in vivo* simulations.

For magnetic sensor noise, to make a fair comparison considering the difference in units—volts vs. tesla—the average signal power across the sensors for the entire recording duration was calculated for each magnetic field component (*B*_*x*_, *B*_*y*_, *B*_*z*_) and the ϕ data. The magnitude of the magnetic field signals was then scaled up so that the average signal power of the magnetic field data matched the average signal power of the voltage data (numerically, ignoring units). After the additive sensor noise was included, each field had an equivalent average SNR. This also ensured that the scale of the signals was approximately identical when fed into the sorting algorithm which was important to minimize unwanted variance. For multimodal measurements, the fields were scaled according to the signal type with the largest average power (so as to preserve the relative magnitudes of each field type).

### 4.4 Simulated spike sorting

Kilosort2 [47] was used within the SpikeInterface [48] framework for spike sorting. The only significant modification we made to the Kilosort algorithm was with regards to spike detection based on threshold crossing: whereas the original algorithm detected only negative spikes, we set it to detect either negative or positive spikes for magnetic fields. Implementing this change for voltage signals worsened the overall performance (the largest spikes, usually near the soma, typically have negative polarity), and so was not included, but it was essential for magnetic fields given that their largest spikes can have different polarities depending on the direction of longitudinal currents relative to the sensor positions.

We performed a grid search across Kilosort2’s detection threshold and minimum spiking frequency variables for each combination of recording parameters—recording duration, number of neurons, noise level, and sensor configuration—to attempt to find the close-to-optimal sorting performance for each parameter set. We randomly generated *N* = 10 recordings for each combination of parameters and then, for each recording, determined the average sorting accuracy, defined as true positive / (true positive + false positive + false negative) for predicted spikes, and the number of well-detected units, defined as having greater than 80% match between their ground-truth spike train and spike-sorted spike train [48]. The highest-accuracy sorts and highest well-detected-count sorts for each signal $s\in \{\phi ,B_{x},B_{y},B_{z}\}$ across the grid search were isolated and compared to get an estimate of the upper bound of sorting performance per field type, regardless of sorting condition. The full sorting pipeline is shown in S8 Fig.

### 4.5 Morphology reconstruction modified algorithm

The CNBE algorithm to enhance the performance of the GNBE approach for morphology reconstruction proceeds as follows. First, a naive SVM boundary is calculated. Next, the location of the axon hillock (the region of the cell typically responsible for the strongest magnetic field signal [67]) is estimated by finding the weighted center of the coordinates of the 4 sensors that measured the signals with the greatest average power. This procedure is repeated for the next 96 highest power sensors, producing 24 points that were estimated to lie close to, or in the general direction of, the neuron’s actual morphology. The 24 points along the naive SVM boundary lying closest to these points are then determined, and “outliers”—with an average distance to their 4 nearest neighbors greater than 50 μm—are removed. A line of best fit that runs through the remainder of these 24 points and the axon hillock coordinate is then calculated (strongly weighted to pass close to the axon hillock). A line perpendicular to this best fit line and lying 10 μm “behind” the axon hillock (the opposite direction of the vector pointing from the axon hillock to the 24 points) is then constructed—any sensor behind this line is then excluded from a second SVM calculation, which produces an updated morphology estimate.

## Supporting information

S1 Appendix Supplementary math. Proofs for Equations 1, 2, and 3 are presented along with an explanation of how neural signals scale in space as a function of the signal type.
(PDF)

S1 FigThe six layer V rat somatosensory cortex neuron models (n=0−5) used in the study.Models obtained from Blue Brain Project (BBP) [68]. Model 0: Double bouquet cell (DBC, BBP ID: L5_DBC_bAC217_1). Model 1: Martinotti cell (MC, BBP ID: L5_MC_bAC217_1). Model 2: Thick-tufted pyramidal cell with a late bifurcating apical tuft (TTPC1, BBP ID: L5_TTPC1_cADpyr232_1). Model 3: Thick-tufted pyramidal cell with an early bifurcating apical tuft (TTPC2, BBP ID: L5_TTPC2_cADpyr232_1). Model 4: Slender-tufted pyramidal cell (STPC, BBP ID: L5_STPC_cADpyr232_1). Model 5: Untufted pyramidal cell (UTPC, BBP ID: L5_UTPC_cADpyr232_1).(TIF)

S2 FigNeural magnetic field scaling for planar vs. perpendicular components.The rotating magnetic field generated around a neuron (cross-section shown) suspended some Δz above an array of sensors points entirely in the planar *B*_*y*_ direction directly underneath the neuron but increasingly points in the normal *B*_*z*_ direction for sensors at distances greater than Δz.(TIF)

S3 FigRadial asymptotic signal scaling.Asymptotic negative scaling exponent −*n* for the extracellular potential $\phi_{e}$ and the magnetic field components *B*_*e*,*y*_ and *B*_*e*,*z*_ for cells 0–5 (S1 Fig). Evaluated between 0–50 μm down the length of the apical dendrite, *N* = 52.(TIF)

S4 FigNeural electrical potential and magnetic field distribution.Normalized magnitude of the peak signal at each point in space around Cell 2 (S1 Fig) over the time duration of a single spike for ϕ, *B*_*x*_, *B*_*y*_, and *B*_*z*_.(TIF)

S5 FigCellular spatial resolution limit study diagram.Neurons are positioned above a dense, simulated array of sensors. Spike templates are generated by translating, $T_{trans}(\Delta {𝐫}_{1}){𝐌}_{n}^{s}$, and rotating, $T_{trans}(\Delta {𝐫}_{2})T_{rot}(\theta ){𝐌}_{n}^{s}$, the neurons within the sensor plane. Displayed are abridged spike templates comprising 24 measurement points for three distinct neuron positions and orientations.(TIF)

S6 FigMulti-source discrimination analysis for all cells and density sweep for Cells 2 and 3.**a**, Negative logarithm of the average condition number for channel matrices constructed from constant-density populations of *N*_*C*_ = 50, 113, and 200 cells (Cells 0–5 from S1 Fig) corresponding to sensor array pitch of 50, 75, and 100 μm, respectively, for signal types $s\in \{\phi ,B_{x}$, *B*_*y*_, *B*_*z*_, (*B*_*x*_, *B*_*y*_), (*B*_*x*_, *B*_*y*_, *B*_*z*_), (ϕ, *B*_*z*_)} (*N* = 30). **b**, Inverse condition number as a function of cell count for arrays with 75-μm pitch, using Cells 2 and 3 from S1 Fig (*N* = 30).(TIF)

S7 Fig Setup for spike sorting simulations. **a**, Simulation setup for planar *in vitro* arrays. The array consists of a 10×10 grid of measurement points, with neurons randomly positioned and oriented above the grid. **b**, Simulation setup for a Neuropixel-style *in vivo* probe. **c**, Diagram of effective radius simulation for *in vivo probe*, with neurons distributed within a defined radius on the front side of the probe.
(TIF)

S8 FigSpike sorting pipeline.First, multiple randomized sets of cell templates are chosen. Each recording will consist of *N*_*C*_ cells. Each cell template is simulated to generate $\phi ,B_{x},B_{y}$, and *B*_*z*_ templates for multiple sensor pitch values or probe configurations. Each set of templates is then convolved with a set of spike trains, and noise is added, with different levels, to generate the final recordings. Each recording is then processed multiple times with Kilosort2 using different parameters each time, and the best sorting performance and accuracy are extracted for each of the different conditions.(TIF)

S9 FigBiological neuron noise simulation.Sorting accuracy as a function of noise neuron count (neurons located between 100–200 μm from the probe) for each signal type (400 signal neurons within 10–100 μm radius, 120 s duration).(TIF)

S1 VideoTime evolution of extracellular signals from an action potential.Extracellular electrical potential ϕ and magnetic field components *B*_*x*_, *B*_*y*_, and *B*_*z*_ plotted for the duration of a spike for Cell 2 (S1 Fig). Each plot is normalized to its maximum absolute value.(MP4)
