# Supplementary material for: Spatial richness of neural magnetic fields
Source: PLoS Comput Biol. 2026 May 22;22(5):e1014283. doi: 10.1371/journal.pcbi.1014283 (PMC13196990; doi:10.1371/journal.pcbi.1014283)
Supplement: S1 Appendix — Proofs for Equations 1, 2, and 3 are presented along with an explanation of how neural signals scale in space as a function of the signal type. (PDF) [file pcbi.1014283.s001.pdf]

Supplementary Information

Ziad Ali[1] and Ada S. Y. Poon[1*]

[1]Electrical Engineering Department, Stanford University, Stanford, California, USA.

*Corresponding author(s). E-mail(s): adapoon@stanford.edu;
Contributing authors: ziadaali@stanford.edu;

S1 Appendix

1 Proof of Equation (1) in Main Text

The vector form of Green's theorem states that

$$\int_{\mathcal{V}} \mathbf{Q} \cdot \nabla \times \nabla \times \mathbf{P} - \mathbf{P} \cdot \nabla \times \nabla \times \mathbf{Q} \, d^3 r = \oint_{\mathcal{S}} (\mathbf{P} \times \nabla \times \mathbf{Q} - \mathbf{Q} \times \nabla \times \mathbf{P}) \cdot \hat{\mathbf{n}} \, da \tag{1}$$

for any well-behaved vector fields $\mathbf{P}$ and $\mathbf{Q}$ defined in the volume $\mathcal{V}$ enclosed by the surface $\mathcal{S}$ [1]. By choosing $\mathbf{P} = \mathbf{H}$ and $\mathbf{Q} = \mathbf{c}/|\mathbf{r} - \mathbf{r}'|$ for any constant vector $\mathbf{c}$ in the vector form of Green's theorem, we obtain

$$\begin{aligned} &\mathbf{c} \cdot \int_{\mathcal{V}} \frac{\nabla' \times \nabla' \times \mathbf{H}(\mathbf{r}')}{|\mathbf{r} - \mathbf{r}'|} d^3 r' - \int_{\mathcal{V}} \mathbf{H}(\mathbf{r}') \cdot \nabla' \times \nabla' \times \frac{\mathbf{c}}{|\mathbf{r} - \mathbf{r}'|} d^3 r' \\ &= \oint_{\mathcal{S}} \mathbf{H}(\mathbf{r}') \times \nabla' \times \frac{\mathbf{c}}{|\mathbf{r} - \mathbf{r}'|} \cdot \hat{\mathbf{n}}' \, da' - \oint_{\mathcal{S}} \left[\frac{\mathbf{c}}{|\mathbf{r} - \mathbf{r}'|} \times \nabla' \times \mathbf{H}(\mathbf{r}') \right] \cdot \hat{\mathbf{n}}' \, da'. \end{aligned} \tag{2}$$

In the second term, we have

$$\nabla' \times \nabla' \times \frac{\mathbf{c}}{|\mathbf{r} - \mathbf{r}'|} = \nabla' \left(\nabla' \cdot \frac{\mathbf{c}}{|\mathbf{r} - \mathbf{r}'|} \right) - \nabla'^2 \frac{\mathbf{c}}{|\mathbf{r} - \mathbf{r}'|} = \nabla' \left(\nabla' \cdot \frac{\mathbf{c}}{|\mathbf{r} - \mathbf{r}'|} \right) + 4\pi \mathbf{c} \, \delta(\mathbf{r} - \mathbf{r}') \tag{3a}$$

and

$$\mathbf{H}(\mathbf{r}')\cdot\nabla'\left(\nabla'\cdot\frac{\mathbf{c}}{|\mathbf{r}-\mathbf{r}'|}\right)=\nabla'\cdot\left[\mathbf{H}(\mathbf{r}')\nabla'\cdot\frac{\mathbf{c}}{|\mathbf{r}-\mathbf{r}'|}\right]=\nabla'\cdot\left[\left(\mathbf{c}\cdot\nabla'\frac{1}{|\mathbf{r}-\mathbf{r}'|}\right)\mathbf{H}(\mathbf{r}')\right]. \tag{4}$$

Substituting into the second term yields

$$\int_{\mathcal{V}}\mathbf{H}(\mathbf{r}')\cdot\nabla'\times\nabla'\times\frac{\mathbf{c}}{|\mathbf{r}-\mathbf{r}'|}\,d^3r'=\int_{\mathcal{S}}\left(\mathbf{c}\cdot\nabla'\frac{1}{|\mathbf{r}-\mathbf{r}'|}\right)\mathbf{H}(\mathbf{r}')\cdot\hat{\mathbf{n}}'\,da'+4\pi\int_{\mathcal{V}}\mathbf{H}(\mathbf{r}')\cdot\mathbf{c}\,\delta(|\mathbf{r}-\mathbf{r}'|)\,d^3r' \tag{5a}$$

$$=\mathbf{c}\cdot\left\{\int_{\mathcal{S}}\nabla'\frac{1}{|\mathbf{r}-\mathbf{r}'|}\left[\mathbf{H}(\mathbf{r}')\cdot\hat{\mathbf{n}}'\right]\,da'+4\pi\int_{\mathcal{V}}\mathbf{H}(\mathbf{r}')\,\delta(|\mathbf{r}-\mathbf{r}'|)\,d^3r'\right\}. \tag{5b}$$

In the third term, we have

$$\mathbf{H}(\mathbf{r}')\times\nabla'\times\frac{\mathbf{c}}{|\mathbf{r}-\mathbf{r}'|}\cdot\hat{\mathbf{n}}'=-\nabla'\times\frac{\mathbf{c}}{|\mathbf{r}-\mathbf{r}'|}\cdot\left[\mathbf{H}(\mathbf{r}')\times\hat{\mathbf{n}}'\right] \tag{6a}$$

$$=\left(\mathbf{c}\times\nabla'\frac{1}{|\mathbf{r}-\mathbf{r}'|}\right)\cdot\left[\mathbf{H}(\mathbf{r}')\times\hat{\mathbf{n}}'\right] \tag{6b}$$

$$=\mathbf{c}\cdot\left\{\nabla'\frac{1}{|\mathbf{r}-\mathbf{r}'|}\times\left[\mathbf{H}(\mathbf{r}')\times\hat{\mathbf{n}}'\right]\right\}. \tag{6c}$$

Finally, the integrand in the fourth term can be written as

$$\left[\frac{\mathbf{c}}{|\mathbf{r}-\mathbf{r}'|}\times\nabla'\times\mathbf{H}(\mathbf{r}')\right]\cdot\hat{\mathbf{n}}'=\mathbf{c}\cdot\left[\frac{\nabla'\times\mathbf{H}(\mathbf{r}')}{|\mathbf{r}-\mathbf{r}'|}\times\hat{\mathbf{n}}'\right]. \tag{7}$$

Putting together, we obtain

$$\mathbf{c}\cdot\left\{4\pi\int_{\mathcal{V}}\mathbf{H}(\mathbf{r}')\delta(\mathbf{r}-\mathbf{r}')\,d^3r'-\int_{\mathcal{V}}\frac{\nabla'\times\nabla'\times\mathbf{H}(\mathbf{r}')}{|\mathbf{r}-\mathbf{r}'|}\,d^3r'-\oint_{\mathcal{S}}\frac{\nabla'\times\mathbf{H}(\mathbf{r}')}{|\mathbf{r}-\mathbf{r}'|}\times\hat{\mathbf{n}}'\,da'\right.$$
$$\left.+\oint_{\mathcal{S}}\nabla'\frac{1}{|\mathbf{r}-\mathbf{r}'|}\left[\mathbf{H}(\mathbf{r}')\cdot\hat{\mathbf{n}}'\right]da'+\oint_{\mathcal{S}}\nabla'\frac{1}{|\mathbf{r}-\mathbf{r}'|}\times\left[\mathbf{H}(\mathbf{r}')\times\hat{\mathbf{n}}'\right]da'\right\}=0. \tag{8}$$

Since (8) is true for all $\mathbf{c}$, the expression inside the curly brackets should be zero, yielding our desired identity.

Referring to Fig 1a, we apply this identify to the combined volume $\mathcal{V}=\mathcal{V}_e\cup\mathcal{V}_i$. Given that the membrane is very thin, $\hat{\mathbf{n}}_i\approx-\hat{\mathbf{n}}_e$ denoted simply by $\hat{\mathbf{n}}$ and $\mathcal{S}_i\approx\mathcal{S}_e$ denoted by $\mathcal{S}$. Additionally, we utilize the relation $\nabla\times\mathbf{H}=\mathbf{J}_{imp}-\sigma\nabla\phi$ where $\mathbf{J}_{imp}$ is the impressed source, ϕ is the electrical potential, and σ is the conductivity. The impressed source is confined entirely within the volume, with no contribution

on the surface. Consequently, for all $\mathbf{r} \in \mathcal{V}_e$,

$$
\begin{aligned}
4\pi \mathbf{H}_e(\mathbf{r}) = &\int_{\mathcal{V}_e} \frac{\nabla' \times \mathbf{J}_{imp}(\mathbf{r}')}{|\mathbf{r}-\mathbf{r}'|} d^3r' - \oint_{\mathcal{S}} \frac{\sigma_i \nabla' \phi_i(\mathbf{r}') \times \hat{\mathbf{n}}'}{|\mathbf{r}-\mathbf{r}'|}\, da' + \oint_{\mathcal{S}} \frac{\sigma_e \nabla' \phi_e(\mathbf{r}') \times \hat{\mathbf{n}}'}{|\mathbf{r}-\mathbf{r}'|}\, da' \\
&- \oint_{\mathcal{S}} \nabla' \frac{1}{|\mathbf{r}-\mathbf{r}'|} \Big\{ \big[\mathbf{H}_i(\mathbf{r}') - \mathbf{H}_e(\mathbf{r}')\big] \cdot \hat{\mathbf{n}}' \Big\}\, da' \\
&- \oint_{\mathcal{S}} \nabla' \frac{1}{|\mathbf{r}-\mathbf{r}'|} \times \Big\{ \big[\mathbf{H}_i(\mathbf{r}') - \mathbf{H}_e(\mathbf{r}')\big] \times \hat{\mathbf{n}}' \Big\}\, da' \qquad (9)
\end{aligned}
$$

where the subscripts e and i denote variables in the extracellular and intracellular spaces, respectively. Due to the membrane's much lower conductivity compared to both σ_e and σ_i, the longitudinal current within the membrane is negligible. Combined with the membrane's small thickness, this results in the magnetic field remaining nearly continuous across the membrane, rendering the last two integrals negligible. Thus, the extracellular magnetic field can be described as the sum of the field originated by the solenoidal component of the impressed source (for example, magnetic stimulation from a microcoil) in an unbounded medium and the field originated by the longitudinal component (orthogonal to $\hat{\mathbf{n}}$) of the current density along the inner and outer surfaces of the cell membrane.

To highlight how the extracellular magnetic field and electrical potential arise from distinct components of the current density on the inner surface of the membrane defined by $\mathbf{i}_{is} = -\sigma_i \nabla \phi_i$ on $\mathcal{S}_i$, we simplify by assuming a source-free and extended extracellular space. In such a space, the longitudinal component of the current density along the outer membrane surface and the extracellular potential at the outer membrane surface become negligible. Furthermore, assuming this space has negligible magnetic properties (magnetization $\mathbf{M} \approx 0$), we can linearly relate the magnetic flux density $\mathbf{B}$ to the magnetic field intensity by $\mathbf{B} = \mu_0 \mathbf{H}$. Consequently,

$$
\mathbf{B}_e(\mathbf{r}) \approx \frac{\mu_0}{4\pi} \oint_{\mathcal{S}} \frac{-\sigma_i \nabla' \phi_i(\mathbf{r}') \times \hat{\mathbf{n}}'}{|\mathbf{r}-\mathbf{r}'|}\, da' \qquad (10)
$$

2 Proof of Equation (2) in Main Text

By applying the scalar form of Green's second identity to $\mathcal{V} = \mathcal{V}_e$, for all $\mathbf{r} \in \mathcal{V}_e$ [2],

$$
4\pi \sigma_e \phi_e(\mathbf{r}) = -\int_{\mathcal{V}_e} \frac{\nabla' \cdot \mathbf{J}_{imp}(\mathbf{r}')}{|\mathbf{r}-\mathbf{r}'|} d^3r' - \oint_{\mathcal{S}} \frac{\sigma_e \nabla' \phi_e(\mathbf{r}') \cdot \hat{\mathbf{n}}'}{|\mathbf{r}-\mathbf{r}'|}\, da' + \oint_{\mathcal{S}} \sigma_e \phi_e(\mathbf{r}') \nabla' \frac{1}{|\mathbf{r}-\mathbf{r}'|} \cdot \hat{\mathbf{n}}'\, da'. \qquad (11)
$$

Given the membrane's small thickness and its conductivity being much lower than both σ_e and σ_i, the transverse current is approximately continuous across the membrane, leading to $-\sigma_e \nabla \phi_e(\mathbf{r}) \cdot \hat{\mathbf{n}} =$

$-\sigma_i \nabla \phi_i(\mathbf{r}) \cdot \hat{\mathbf{n}}$ on $\mathcal{S}$. As a result, the extracellular potential can be described as the sum of the field originated by the irrotational component of the impressed source (for example, electrical stimulation from microelectrodes) in an unbounded medium and the field originated by the transverse component (parallel to $\hat{\mathbf{n}}$) of the current density crossing the inner or outer surfaces of the cell membrane. If we assume a source-free and extended extracellular space where the extracellular potential at the outer membrane surface becomes negligible, we obtain

$$\phi_e(\mathbf{r}) \approx \frac{1}{4\pi\sigma_e} \oint_{\mathcal{S}} \frac{-\sigma_i \nabla' \phi_i(\mathbf{r}') \cdot \hat{\mathbf{n}}'}{|\mathbf{r} - \mathbf{r}'|} \, da'. \tag{12}$$

3 Proof of Equation (3) in Main Text

To analyze the sign reversal, we model the neuron as an infinitely long cylindrical axon embedded in an unbounded volume conductor. Solutions to Laplace's equation in cylindrical coordinates are expressed using modified Bessel functions [3], yielding the intracellular and extracellular potentials as

$$\Phi_i(\rho, k) = A_i(k) I_0(|k|\rho) \tag{13a}$$

$$\Phi_e(\rho, k) = A_e(k) K_0(|k|\rho). \tag{13b}$$

Here, $\Phi_{i/e}(\rho, k)$ denotes the Fourier transform of $\phi_{i/e}(\rho, z)$ defined as $\Phi_{i/e}(\rho, k) = \int_{-\infty}^{\infty} \phi_{i/e}(\rho, z) e^{-jkz} \, dz$, $I_n(\cdot)$ and $K_n(\cdot)$ are modified Bessel functions of the first and second kind of order n, respectively, and $A_{i/e}(k)$'s are functions determined by boundary conditions. To capture the neuron's activity during firing, we follow the approach outlined in Refs. 4–6. We determine $A_{i/e}(k)$'s such that solutions to the Laplace's equation satisfy a given transmembrane potential, $\phi_m(z)$, representing the action potential for an active neuron. As a result, we get

$$\Phi_i(\rho, k) = \frac{\frac{I_0(|k|\rho)}{I_0(|k|a)}}{1 + \frac{\sigma_i}{\sigma_e} \frac{I_1(|k|a)}{I_0(|k|a)} \frac{K_0(|k|a)}{K_1(|k|a)}} \Phi_m(k) \tag{14}$$

where a is the radius of the cylindrical axon. Given that the length of the axon is much greater than its radius, the characteristic length scale over which the transmembrane potential has significant values, denoted by L, is expected to be much larger than the radius of the axon, that is, $L \gg a$. As a result, the Fourier transform of the transmembrane potential, $\Phi_m(k)$, becomes negligible for $|k|$ greater than a few multiples of $1/L$. Thus, it is reasonable to assume that $|k|a \ll 1$ and the modified Bessel functions can

be approximated as follows:

$$I_0(|k|a) \approx 1 \qquad I_1(|k|a) \approx \frac{|k|a}{2} \tag{15a}$$

$$K_0(|k|a) \approx -\ln(|k|a) \qquad K_1(|k|a) \approx \frac{1}{|k|a}. \tag{15b}$$

Applying these approximations, Equation (14) simplifies to

$$\Phi_i(\rho,k) \approx \frac{I_0(|k|\rho)}{1 - \frac{\sigma_i}{2\sigma_e}(|k|a)^2 \ln(|k|a)}\,\Phi_m(k). \tag{16}$$

Since $\lim_{x\to 0} x^2 \ln x = 0$, this reduces further to

$$\Phi_i(\rho,k) \approx I_0(|k|\rho)\,\Phi_m(k). \tag{17}$$

The longitudinal and the transmembrane components of the intracellular surface current density are given by

$$i^l_{is,z}(z) = -\sigma_i \frac{\partial \phi_i(\mathbf{r})}{\partial z}\bigg|_{\rho=a} \quad \text{and} \quad i^t_{is,\rho}(z) = -\sigma_i \frac{\partial \phi_i(\mathbf{r})}{\partial \rho}\bigg|_{\rho=a}, \tag{18}$$

respectively. Applying the Fourier transform along z, we obtain

$$I^l_{is,z}(k) = -\sigma_i \cdot ik\Phi_i(a,k) \quad \text{and} \quad I^t_{is,\rho}(k) = -\sigma_i \frac{\partial \Phi_i(\rho,k)}{\partial \rho}\bigg|_{\rho=a}, \tag{19}$$

respectively. From Equation (17), we obtain

$$I^l_{is,z}(k) \approx -jk\sigma_i I_0(|k|a)\,\Phi_m(k) \quad \text{and} \quad I^t_{is,\rho}(k) \approx -\sigma_i|k|I_0'(|k|a)\,\Phi_m(k), \tag{20}$$

As $|k|a \ll 1$, $I_0(|k|a) \approx 1$ and $I_0'(|k|a) = I_1(|k|a) \approx \frac{|k|a}{2}$. This yields Equation (3) in the main text.

4 Explanation of Neural Signal Scaling

First, we need to define the region of operation in biological tissue. At 1 kHz or below, $\omega\epsilon/\sigma < 0.08$ in tissue, implying that the medium is a good conductor [7]. Volume charges therefore disappear quickly, implying that $\rho = 0$. The analysis is similar to a steady current in a conducting medium which produces a static magnetic field and a static electric field. The electric and magnetic fields satisfy Poisson's equation.

For any impressed current source $\mathbf{J}_{imp}$, we can also express it in terms of impressed charges $\rho_{imp} = \frac{1}{i\omega}\nabla \cdot \mathbf{J}_{imp}$. In a uniform isotropic medium, Poisson's equation yields

$$\nabla^2\phi = -\frac{\rho_{imp}}{\epsilon - \frac{\sigma}{i\omega}} = \frac{-\nabla \cdot \mathbf{J}_{imp}}{\sigma - i\omega\epsilon}. \tag{21}$$

As the medium is a good conductor ($\omega\epsilon << \sigma$),

$$\nabla^2\phi = -\frac{-\nabla \cdot \mathbf{J}_{imp}}{\sigma} = -\frac{I_v}{\sigma} \tag{22}$$

where $I_v = -\nabla \cdot \mathbf{J}_{imp}$, representing the spatial distribution of *monopolar* current sources and sinks, each of which produces an electric field that scales as $1/R^2$. Note that I_v is distinct from the current source density $\mathbf{J}_{imp}$ used in the Biot-Savart Law [8].

The magnetic field from the axial current $\mathbf{J}_{imp}$ scales as $1/R^2$. It is important to note that while the axial currents are *current* dipoles, they are not *magnetic* dipoles in isolation; magnetic dipoles are current loops and must therefore include extracellular return currents. However, these currents are so small due to their distribution in the extracellular volume as to be omitted from the calculation (as supported in [9]). This scaling trend is reflected in the well known $\sim 1/R^2$ scaling behavior of finite-lengths of wire as well as the $1/R$ scaling achieved by infinitely long and straight wires.

Therefore, each individual source (transmembrane or axial current) generates a field with $1/R^2$ scaling behavior. The addition of an opposite polarity source — for either transmembrane or axial currents — therefore creates a dipole, a third source creates two opposing dipoles, or a quadropole, and so on. To determine the order of the multipolar field generated by transmembrane or axial currents, to first-order, we therefore only need to determine the number of sign flips of the sources along the cell.

References

[1] Stratton, J. A. *Electromagnetic theory* Reissued ed edn. IEEE Press series on electromagnetic wave theory (IEEE Press [u.a.], Piscataway, NJ, 2007).

[2] Geselowitz, D. B. On Bioelectric Potentials in an Inhomogeneous Volume Conductor. *Biophysical journal* **7**, 1 – 11 (1967).

[3] Jackson, J. D. *Classical electrodynamics* 3. ed., [nachdr.] edn (Wiley, Hoboken, NY, 2009).

[4] Clark, J. & Plonsey, R. A Mathematical Evaluation of the Core Conductor Model. *Biophysical journal* **6**, 95 – 112 (1966).

[5] Geselowitz, D. B. Comment on the Core Conductor Model. *Biophysical journal* **6**, 691 – 692 (1966).

[6] Clark, J. & Plonsey, R. The Extracellular Potential Field of the Single Active Nerve Fiber in a Volume Conductor. *Biophysical journal* **8**, 842 – 864 (1968).

[7] Gabriel, S., Lau, R. W. & Gabriel, C. The dielectric properties of biological tissues: III. parametric models for the dielectric spectrum of tissues. *Phys. Med. Biol.* **41**, 2271–2293 (1996).

[8] Zangwill, A. *Modern Electrodynamics* (Cambridge Univ. Press, Cambridge, 2013).

[9] Woosley, J. K., Roth, B. J. & Wikswo, J. P., Jr. The magnetic field of a single axon: A volume conductor model. *Math. Biosci.* **76**, 1–36 (1985).

[10] Ramaswamy, S. *et al.* The neocortical microcircuit collaboration portal: a resource for rat somatosensory cortex. *Front. Neural Circuits* **9**, 44 (2015).
