# Supplementary figures and images for: Spatial richness of neural magnetic fields
Source: PLoS Comput Biol. 2026 May 22;22(5):e1014283. doi: 10.1371/journal.pcbi.1014283 (PMC13196990; doi:10.1371/journal.pcbi.1014283)

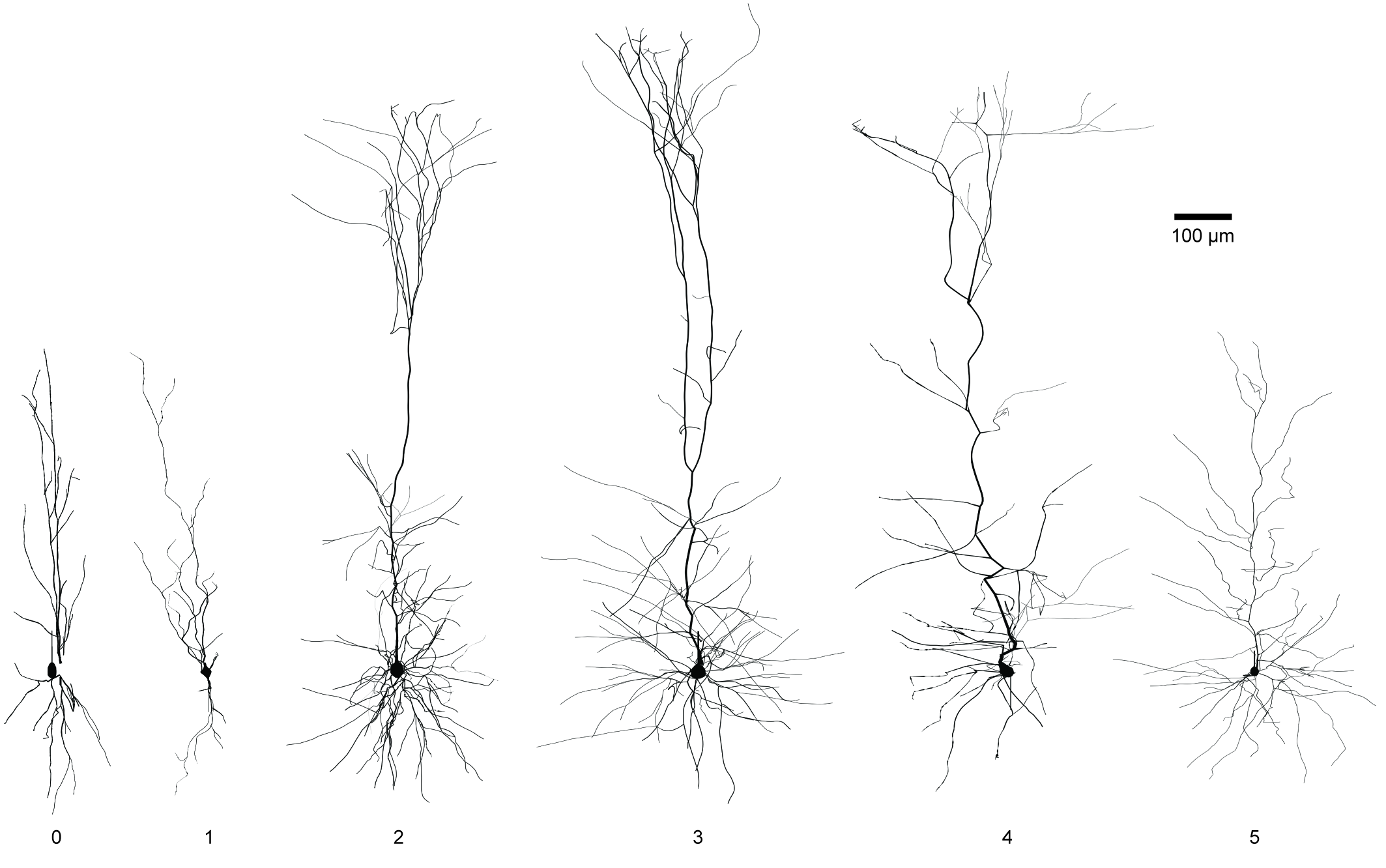

Supplement: S1 Fig — Models obtained from Blue Brain Project (BBP) [68]. Model 0: Double bouquet cell (DBC, BBP ID: L5_DBC_bAC217_1). Model 1: Martinotti cell (MC, BBP ID: L5_MC_bAC217_1). Model 2: Thick-tufted pyramidal cell with a late bifurcating apical tuft (TTPC1, BBP ID: L5_TTPC1_cADpyr232_1). Model 3: Thick-tufted pyramidal cell with an early bifurcating apical tuft (TTPC2, BBP ID: L5_TTPC2_cADpyr232_1). Model 4: Slender-tufted pyramidal cell (STPC, BBP ID: L5_STPC_cADpyr232_1). Model 5: Untufted pyramidal cell (UTPC, BBP ID: L5_UTPC_cADpyr232_1). (TIF) [file pcbi.1014283.s002.tif]

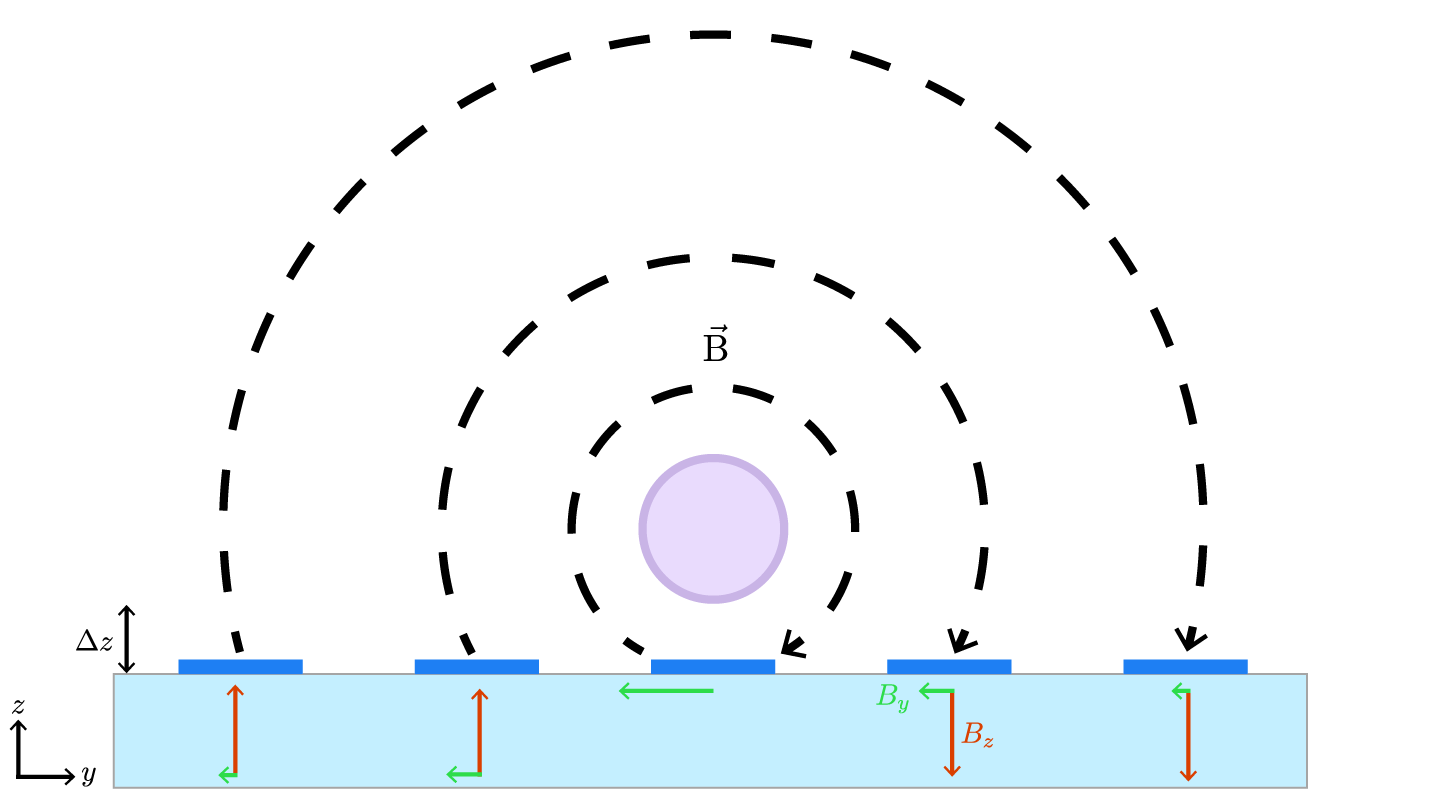

Supplement: S2 Fig — The rotating magnetic field generated around a neuron (cross-section shown) suspended some Δz above an array of sensors points entirely in the planar By direction directly underneath the neuron but increasingly points in the normal Bz direction for sensors at distances greater than Δz. (TIF) [file pcbi.1014283.s003.tif]

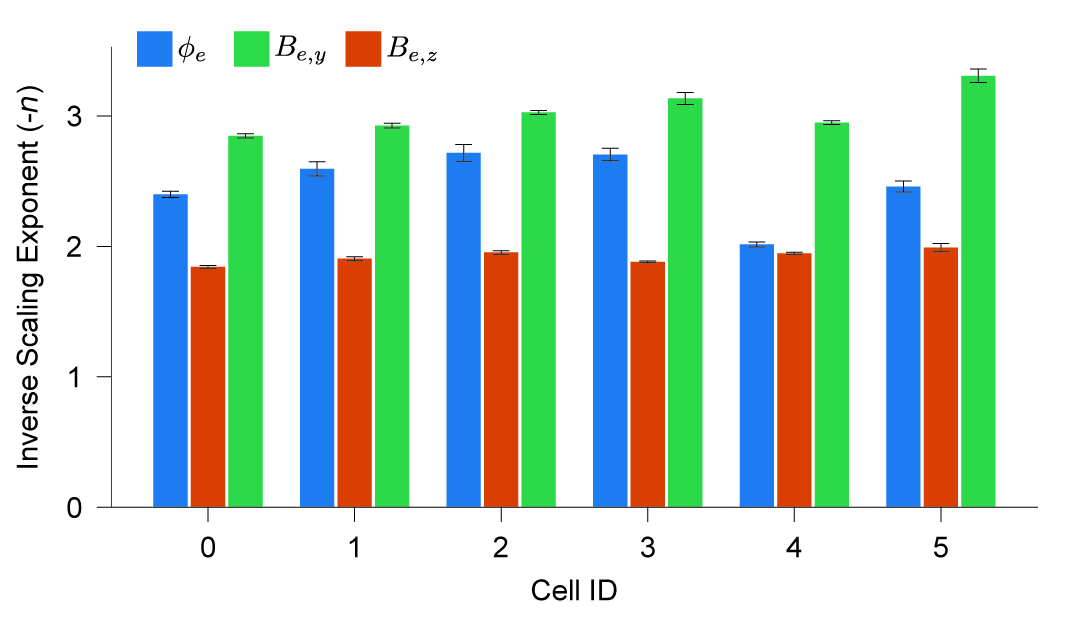

Supplement: S3 Fig — Asymptotic negative scaling exponent −n for the extracellular potential ϕe and the magnetic field components Be,y and Be,z for cells 0–5 (S1 Fig). Evaluated between 0–50 μm down the length of the apical dendrite, N = 52. (TIF) [file pcbi.1014283.s004.tif]

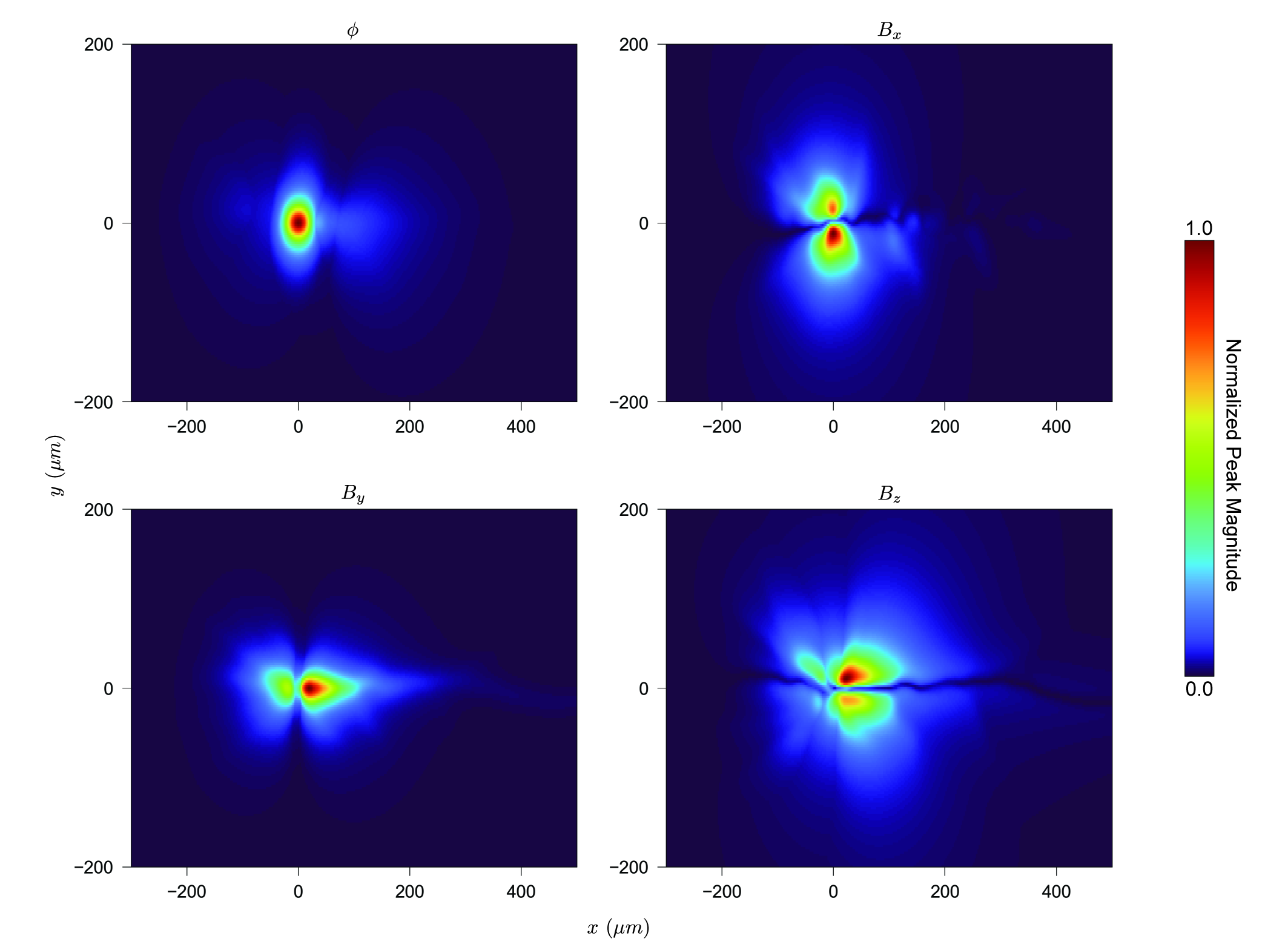

Supplement: S4 Fig — Normalized magnitude of the peak signal at each point in space around Cell 2 (S1 Fig) over the time duration of a single spike for ϕ, Bx, By, and Bz. (TIF) [file pcbi.1014283.s005.tif]

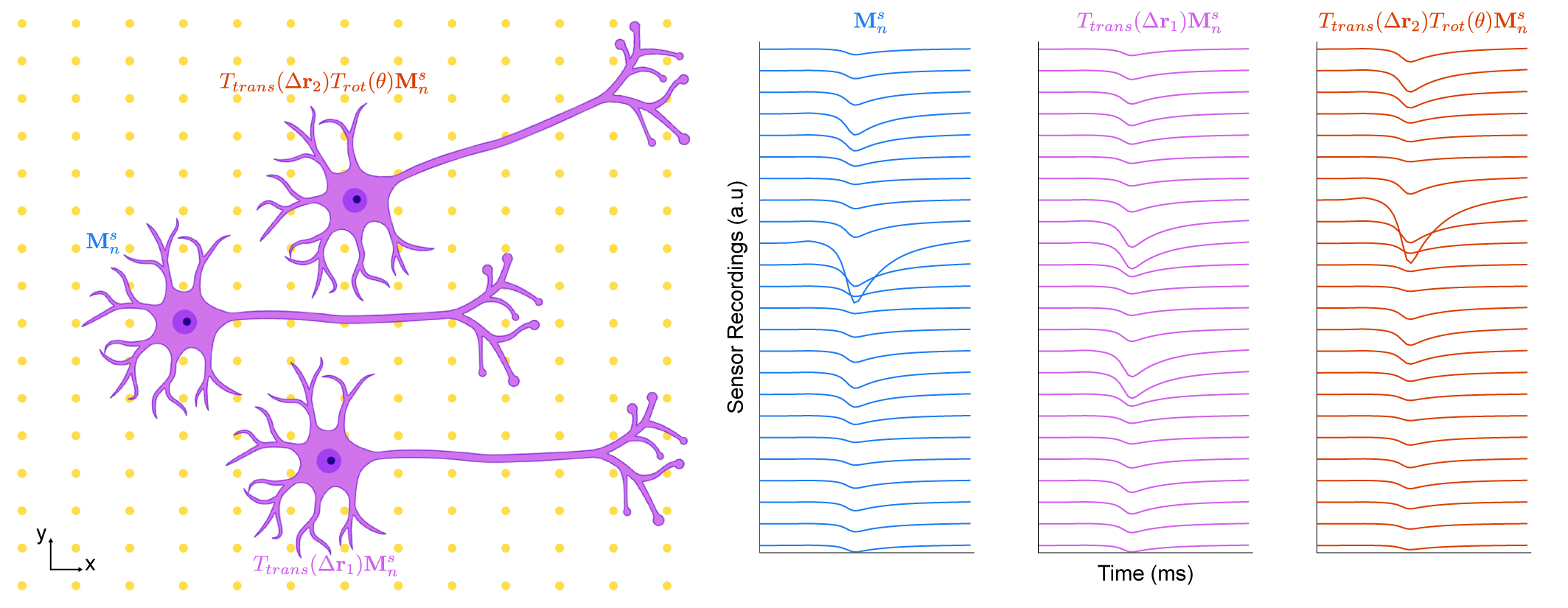

Supplement: S5 Fig — Neurons are positioned above a dense, simulated array of sensors. Spike templates are generated by translating, Ttrans(Δ𝐫1)𝐌ns, and rotating, Ttrans(Δ𝐫2)Trot(θ)𝐌ns, the neurons within the sensor plane. Displayed are abridged spike templates comprising 24 measurement points for three distinct neuron positions and orientations. (TIF) [file pcbi.1014283.s006.tif]

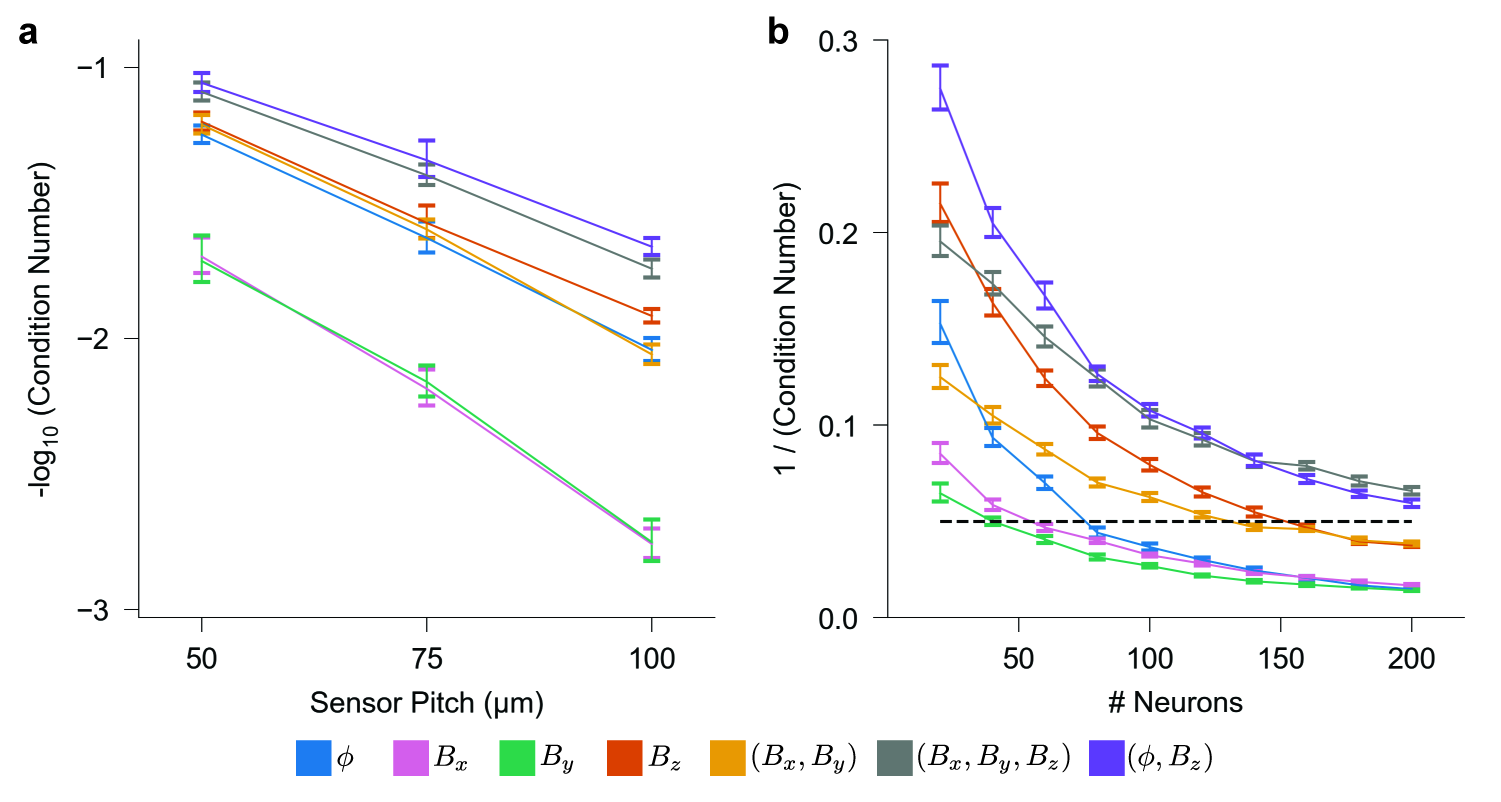

Supplement: S6 Fig — a, Negative logarithm of the average condition number for channel matrices constructed from constant-density populations of NC = 50, 113, and 200 cells (Cells 0–5 from S1 Fig) corresponding to sensor array pitch of 50, 75, and 100 μm, respectively, for signal types s∈{ϕ,Bx, By, Bz, (Bx, By), (Bx, By, Bz), (ϕ, Bz)} (N = 30). b, Inverse condition number as a function of cell count for arrays with 75-μm pitch, using Cells 2 and 3 from S1 Fig (N = 30). (TIF) [file pcbi.1014283.s007.tif]

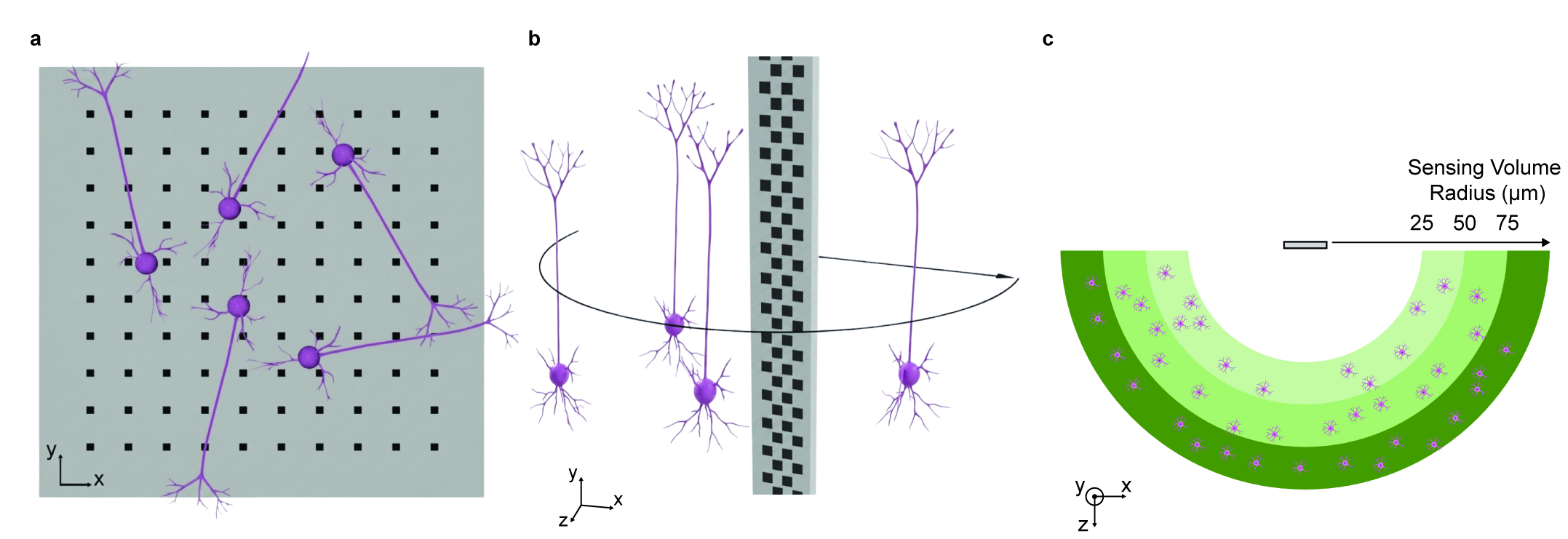

Supplement: S7 Fig — a, Simulation setup for planar in vitro arrays. The array consists of a 10×10 grid of measurement points, with neurons randomly positioned and oriented above the grid. b, Simulation setup for a Neuropixel-style in vivo probe. c, Diagram of effective radius simulation for in vivo probe, with neurons distributed within a defined radius on the front side of the probe. (TIF) [file pcbi.1014283.s008.tif]

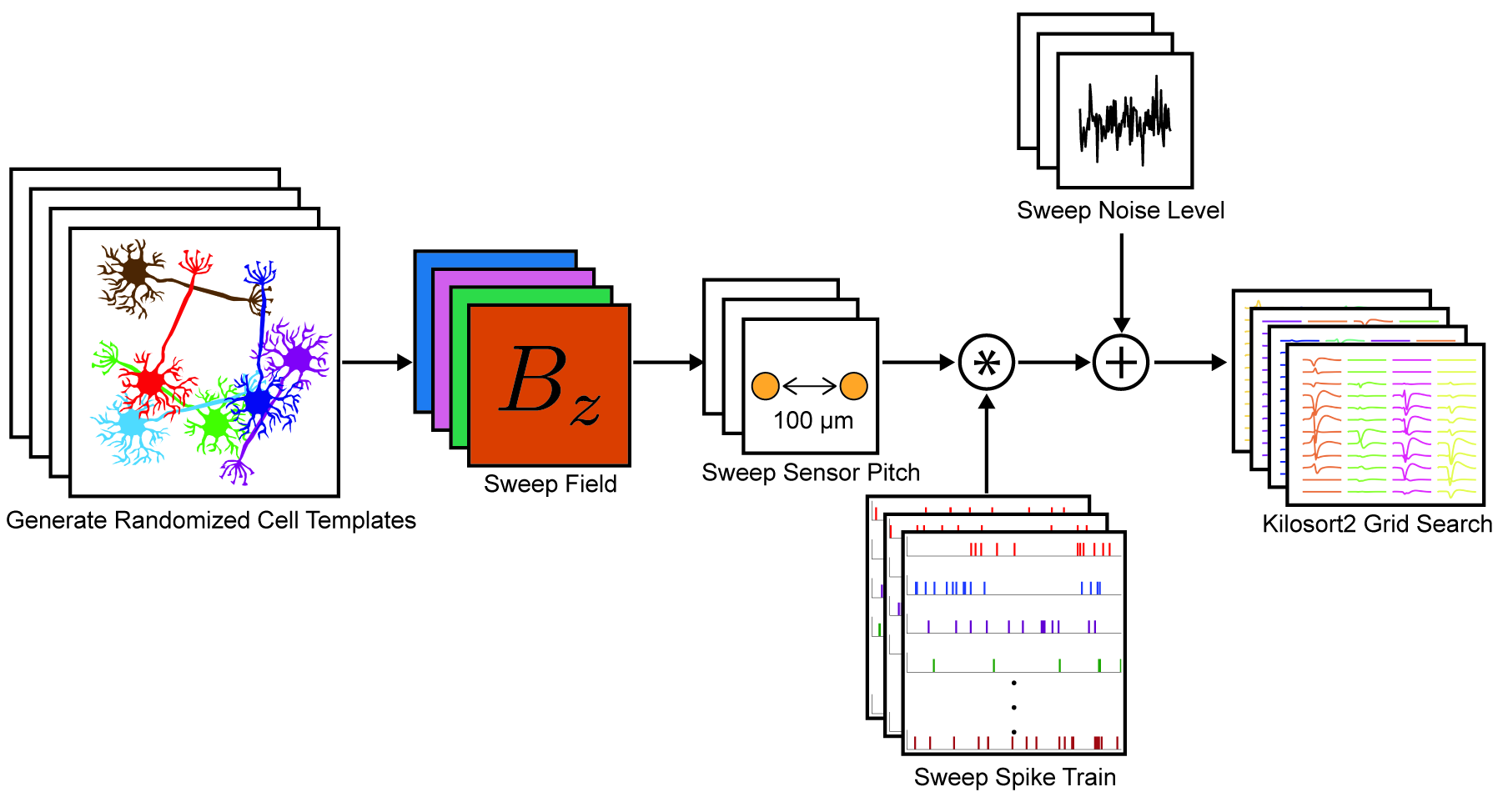

Supplement: S8 Fig — First, multiple randomized sets of cell templates are chosen. Each recording will consist of NC cells. Each cell template is simulated to generate ϕ,Bx,By, and Bz templates for multiple sensor pitch values or probe configurations. Each set of templates is then convolved with a set of spike trains, and noise is added, with different levels, to generate the final recordings. Each recording is then processed multiple times with Kilosort2 using different parameters each time, and the best sorting performance and accuracy are extracted for each of the different conditions. (TIF) [file pcbi.1014283.s009.tif]

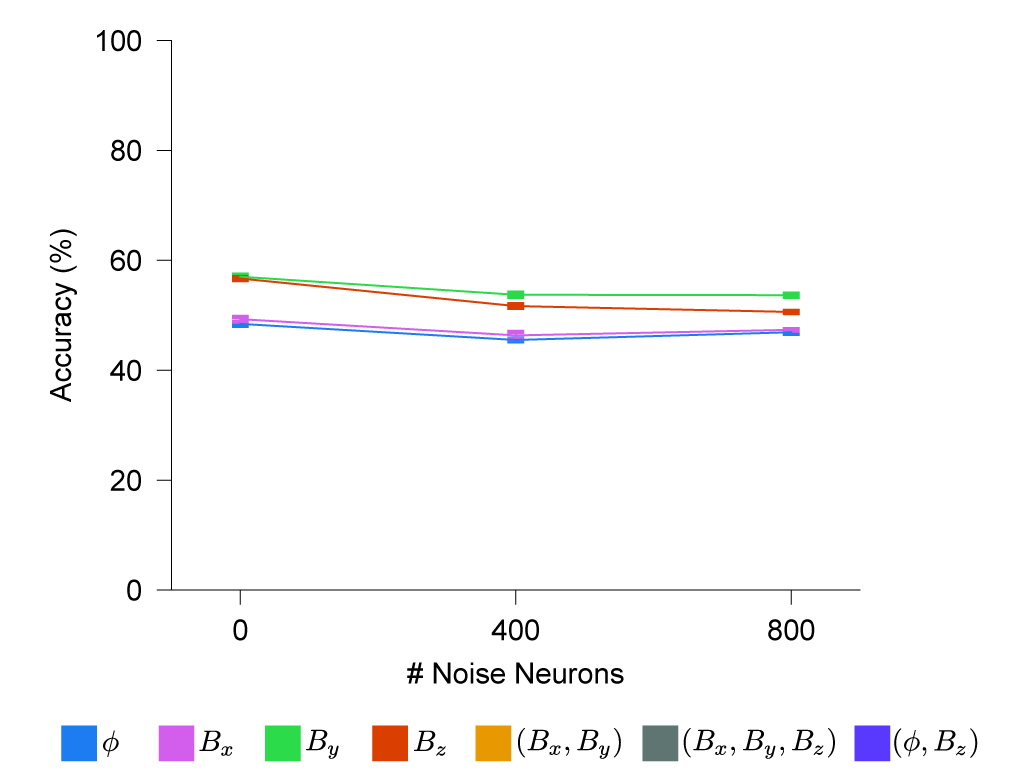

Supplement: S9 Fig — Sorting accuracy as a function of noise neuron count (neurons located between 100–200 μm from the probe) for each signal type (400 signal neurons within 10–100 μm radius, 120 s duration). (TIF) [file pcbi.1014283.s010.tif]
